# Concomitant Processing of Choice and Outcome in Frontal Corticostriatal Ensembles Correlates with Performance of Rats

**DOI:** 10.1093/cercor/bhab091

**Published:** 2021-04-29

**Authors:** Takashi Handa, Rie Harukuni, Tomoki Fukai

**Affiliations:** Department of Behavior and Brain Organization, Center Advanced European Study and Research (Caesar), Bonn 53175, Germany; Department of Neurobiology, Graduate School of Biomedical and Health Sciences (Medicine), Hiroshima University, Hiroshima 734-8553, Japan; Laboratory for Neural Coding and Brain Computing, RIKEN Center for Brain Science, Wako, Saitama 351-0198, Japan; Laboratory for Neural Coding and Brain Computing, RIKEN Center for Brain Science, Wako, Saitama 351-0198, Japan; Laboratory for Neural Coding and Brain Computing, RIKEN Center for Brain Science, Wako, Saitama 351-0198, Japan; Neural Coding and Brain Computing Unit, Okinawa Institute of Science and Technology, Okinawa 904-0495, Japan

**Keywords:** frontal corticostriatal ensemble, Linear Fisher’s discriminant, multi-neuron recordings, population coding, win-stay lose-shift

## Abstract

The frontal cortex-basal ganglia network plays a pivotal role in adaptive goal-directed behaviors. Medial frontal cortex (MFC) encodes information about choices and outcomes into sequential activation of neural population, or neural trajectory. While MFC projects to the dorsal striatum (DS), whether DS also displays temporally coordinated activity remains unknown. We studied this question by simultaneously recording neural ensembles in the MFC and DS of rodents performing an outcome-based alternative choice task. We found that the two regions exhibited highly parallel evolution of neural trajectories, transforming choice information into outcome-related information. When the two trajectories were highly correlated, spike synchrony was task-dependently modulated in some MFC-DS neuron pairs. Our results suggest that neural trajectories concomitantly process decision-relevant information in MFC and DS with increased spike synchrony between these regions.

## Introduction

Deciding actions based on the outcome of past actions is crucial for the survival of animals. The frontal cortex-basal ganglia network is engaged in various forms of decision making involved in adaptive goal-directed behaviors (Nambu 2008; Balleine and O'Doherty 2010; Hikosaka and Isoda 2010; Friedman et al. 2015). These studies have shown how choice- and/or outcome-related information is processed in the frontal cortex and the basal ganglia. However, how the cortico-basal ganglia network encodes behavioral information at the neural ensemble level remains largely unknown. How the related brain regions communicate such information during outcome-based action selection is yet to be clarified (Brown et al. 2004). In this study, we explore whether and how task-related neural activity is coordinated between the medial frontal cortex (MFC) and dorsal striatum (DS), which is a major input structure of the basal ganglia (Wilson 1987; Cheatwood et al. 2003; Reep et al. 2003), of rats performing an outcome-based alternative choice task.

The MFC is engaged in encoding reward and error signals (Narayanan et al. 2013; Hyman et al. 2017) and hence is considered to play a role in monitoring positive and negative outcomes from an action. In particular, the rostral agranular medial cortex or the secondary motor cortex of rodent has been implicated in sensory-cued (Erlich et al. 2011; Handa et al. 2017; Kurikawa et al. 2018) and outcome-based action selection (Sul et al. 2011; Gremel and Costa 2013a). Along the frontal cortico-basal ganglia axis, excitatory MFC outputs project monosynaptically to DS (Wilson 1986; McGeorge and Faull 1989), which also receives glutamatergic inputs from some thalamic nuclei (Cheatwood et al. 2003; Smith et al. 2004) and is modulated by midbrain dopaminergic inputs conveying outcome information (Wickens et al. 2003) and reward prediction error (Schultz 2006). Accordingly, DS is thought to associate a specific action with the resultant outcome (Reynolds et al. 2001; Lauwereyns et al. 2002; Isomura et al. 2013; Nonomura et al. 2018). Consistent with this, lesions in MFC (Sul et al. 2011; Gremel and Costa 2013a) and DS (Packard et al. 1989; Skelin et al. 2014) impair outcome-based choice behaviors.

Evidence from MFC, or more specifically posterior parietal cortex (PPC) and orbitofrontal cortex (OFC), shows that sequential activation of cortical neuron ensembles, which is termed neural trajectory, underlies decision making (Harvey et al. 2012; Bakhurin et al. 2017; Handa et al. 2017). We explore how this trajectory information is conveyed to striatal neurons during decision making. If the role of the striatum is selection of cortical inputs for action generation, as conventionally thought, such selection may occur sparsely in time, making continuously evolving trajectories less relevant to striatal functions. We therefore clarify to what extent cortical and striatal neuron ensembles are coherently activated during decision making. We hypothesize that neural ensembles in the MFC and DS utilize synchronized spikes to communicate with one another during adaptive behavior. Such spikes are ubiquitous in cortical circuits (Salinas and Sejnowski 2001; Buzsáki and Schomburg 2015) and were suggested to enhance information transmission and association between directly/indirectly interconnected brain regions (Koralek et al. 2013; Girardeau et al. 2017).

We trained head-restrained rats to lick either left or right spouts for earning reward (positive outcome) and simultaneously recorded multi-neuron activity from the MFC and DS of these rats. Because a rewarded spout was changed intermittently, the rats had to switch their choice responses to maximize reward, exhibiting behavioral responses similar to a typical win-stay lose-shift behavior. Unexpectedly, we found that neural trajectories emerge and evolve highly coherently in both MFC and DS during a period ranging from choice selection to outcome evaluation of the alternative choice task. In some MFC-DS neuron pairs, the number of coincident spikes was modulated by task events when the trajectory evolution displayed an enhanced coherence. Our results suggest that the adaptive control of outcome-based decision making relies on the coevolution of neural trajectories in MFC and DS and that spike synchrony plays a role in this temporal coordination.

## Materials and Methods

### Animal Preparation

All experiments were approved and carried out in accordance with the Animal Experiment Plan by the Animal Experiment Committee of RIKEN. Male Long-Evans rats (*N* = 34, 6 weeks, 200–220 g, Japan SLC, Inc.) were used. Home cages were set in a temperature and humidity controlled environment with lights maintained on a 12-h light/dark cycle. Prior to a primary surgery, rats were handled briefly and habituated to a stainless-steel cylinder in the cages. Animals underwent three surgical procedures. All surgical procedures were operated under sterile circumstances. Rats were anesthetized with 2% isoflurane. Their body temperature was monitored with a rectal probe and maintained at ~37°C on a heating pad during the surgery.

At a primary surgery, a sliding head-attachment (Narishige, Tokyo, Japan) was implanted on the skull with dual-curing resin cement (Panavia, Kuraray Noritake Dental, Tokyo Japan) and dental resin (Unifast II, GC Co., Tokyo, Japan) as done before in our laboratory (Isomura et al. 2009, 2013; Handa et al. 2017; Kurikawa et al. 2018). Reference and grounding electrodes (teflon-coated silver wire, A-M systems, Washington, USA) were placed on dura mater above the cerebellum. After recovery from the surgery, the rats were deprived water intake in the home cages in order to utilize water as a reward for the execution of a task, although food was available *ad libitum*. Water (10 mL) was supplemented at every weekend. At a second surgery after 19th training session (3 days prior to a first electrophysiological recording session), we injected a retrograde tracer Fluoro-Gold (Fluorochrome, Colorado, USA) into the DS in order to confirm if our recording sites corresponded to the MFC sending corticostriatal projection to the DS. A glass micropipette filled with 2% Fluoro-Gold dissolved in 0.1 M of cacodylic acid was installed on a micromanipulator angled medially by 27°. The pipette was inserted through a tiny burr hole drilled in the skull over left hemisphere (+1.5 mm to Bregma, 1.0 mm lateral to midline, 4.3 mm traveling distance) so that the pipette tip reached the dorsocentral part of striatum (+1.5 mm anterior to Bregma, approximately 3.0 mm lateral to midline, approximately 3.8 mm ventral to pia mater), which rostral agranular medial cortex (AGm) pyramidal neurons directly innervate. Fluoro-Gold was iontophoretically loaded through 7 s pulse of +5.0 μA by 7 s interval for 30 or 60 minutes with an iontophoresis pump (BAB-501, Kation Scientific, Minnesota, USA). Then, the tiny hole was covered with sterilized spongel and dental silicone sealants (Shofu, Kyoto, Japan). At a third surgery after the 21st training session, two cranial windows (1.2 mm diameter) were made above the DS and MFC (i.e., AGm) of left hemisphere (+1.0 and + 3.0 mm to Bregma, 3.0 and 1.0 mm to midline for DS and rostral AGm, respectively) and then its dura maters were removed for electrophysiological recordings. For bilateral muscimol injection session, another tiny burr hole was additionally drilled in the skull above MFC of right hemisphere (+3.0 mm to Bregma, 1.0 mm to midline). The cranial windows were covered with the silicone sealants.

### Behavioral Training Apparatus

**Figure 1:**
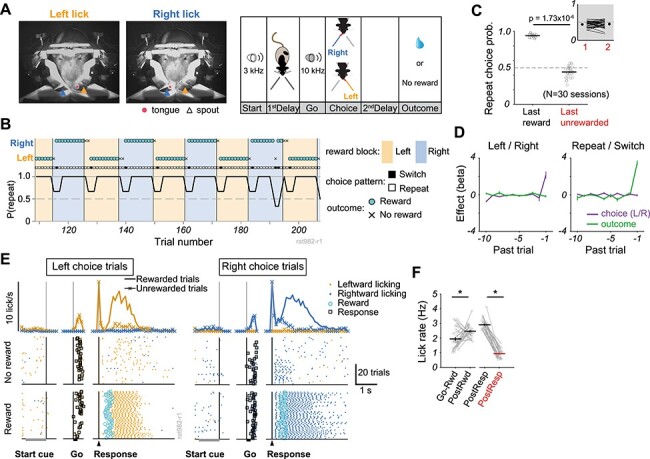
Adaptive choice patterns in response to change in choice-reward contingency. (*A*) *left and middle*: Snapshots of a head-restrained rat making lick responses towards Left (orange arrowhead) and Right (blue arrowhead) spouts. A circle indicates the tongue position. *Right*: A schematic illustration of an outcome-based two alternative choice task. Individual trial started with “Start” tone (3 kHz) presentation. Head-fixed rats were required to wait for “Go” tone (10 kHz) without a lick during “1st Delay” period, and then to make a choice response by licking either one of spouts within 5 s after “Go” tone onset. Reward was given after “2nd Delay” of various durations (0.3–0.7 s) if the chosen position corresponded to current reward location. Otherwise no reward, no sensory feedback, and timeout of 5 s were given. (*B*) Representative flexible choice behavior by a rat across 9 block-reversals of reward position. Vertical lines and colored areas denote the block-reversal and reward position in each block, respectively. Circle and cross indicate the outcome, reward and no-reward respectively, after choice response (Left or Right). Square shows choice pattern (Switch or Repeat). (*C*) Probability of repeating choice after rewarded and unrewarded outcomes (30 sessions, 20 rats). Circle indicates individual sessions. Horizontal lines and error bars represent mean and s.e.m., respectively. The statistical significance was confirmed by Wilcoxon signed-rank test. Inset: Probability of repeating choice following one or two consecutive unrewarded trials. (*D*) Effects of past choice (purple) and outcome (green) on (*left*) current choice position and (*right*) current choice type (repeat and switch). The ordinate shows averaged coefficients of a logistic regression model (*N* = 30 sessions). Error bars show s.e.m. (*E*) Licking patterns during rewarded and unrewarded trials in the session shown in B. Rastergrams denote licking times and locations (Left lick: orange, Right lick: blue). Histograms show averaged Left and Right licking rates in rewarded (solid line) and unrewarded (line with cross) trials. (*F*) Mean licking rate in an epoch between Go tone onset and reward delivery (Go-Rwd), during outcome period of 4 s after reward delivery (PostRwd), and for 4 s after response in rewarded (PostResp in black) and unrewarded (PostResp in red) trials. Asterisks indicate statistical significance (Wilcoxon signed-rank test, *P* < 0.05). Error bars show s.e.m.

Rats were trained to perform a behavioral task controlled under a customized multiple-rats training system (O’hara&Co., Tokyo, Japan), which enabled training several rats to learn a task paradigm in parallel (Isomura et al. 2009, 2013). The behavioral task was controlled by a custom-written software with LabVIEW (National Instruments). In an isolated training chamber, each rat was placed at a body-supporting cylinder, and its head was fixed rigidly and painlessly by screwing a sliding head-holder on a stereotaxic frame. Auditory stimuli were presented at 60-dB SPL via a speaker placed in front of the head-restrained rat. The timing and direction of licking movement were detected when its tongue interrupted an infrared beam placed below the left and right spouts. The detection sensitivity was much enough to detect tongue movements during licking spout, but not sensitive enough to detect other potential movements such as whisking and mastication. White plastic plates were located at both besides of the face in order to prohibit rats from approaching the beam detectable spaces by forelimb reaching (Fig. 1*A*). Spouts were connected to a syringe set on a single-syringe pump (AL-1000, World Precision Instruments, Florida, USA) via silicon tubing. Water delivery from each spout was regulated by an audible pinch valve triggered by TTL signal which also triggered the syringe pumping.

### Behavioral Task

A trial began with a pure tone presentation (3 kHz, 1 s, Start signal) followed by pseudorandom delay period ranged between 0.7–2.3 s (Fig. 1*A*). Within this delay period, rats were required not to lick any spouts from the initial 0.3 s until the appearance of another auditory cue (10 kHz, 0.2 s, Go signal). If the rats licked any spout during this period, the trial was immediately aborted. After the Go signal onset, the rats were allowed to lick one of either left or right spout within a response window (5 s). A first lick was judged as a choice response (Choice). When the chosen spout location corresponded to ongoing reward location, 0.1% saccharin water (15 μL for 0.15 s) was delivered as a reward after a pseudorandom delay period ranging between 0.3 and 0.7 s (second Delay). After an outcome period (4 s, Outcome), a next trial began. On the other hand, when the rats chose no-reward spout, they did not receive any sensory feedback but had an additional time-out of 5 s after the outcome period. After the time-out, a next trial began. Once accumulated total number of rewarded trials reached 10 within each block, the reward-associated spout position was reversed without any feedback like sensory or physical differences in the task. If rats often repeated rewarded choice, the block reversal occurred after around 11–12 trials. If unrewarded choice increased within a block, the block reversal occurred through much more trials. Therefore, rats could not know the block reversal *a priori* without experiencing forthcoming trials (Fig. S2*A*).

### Training

Rats were trained to perform the outcome-based choice task through 21 training sessions. After recovery from the first surgery, at a first training session, the rats learned to associate licking a single spout located at left (or right) side with a reward delivery. Once the rats voluntarily licked the spout to acquire a reward, they learned to associate licking another spout set at the opposite side with a reward acquisition through another spout. At this stage, the second delay period after choice was fixed as zero (no delay). At a second training session, the reward-associated spout location was switched after a bunch of accumulated total rewarded trials were observed at each side. Afterwards, as rats reliably showed repetitive rewarded choices rather than random choices, the number of reversals of reward-location increased. Eventually, we set a block reversal condition that rewarded spout location was reversed after accumulated total number of rewarded trials reached 10 within each block. From 13th and 17th sessions, the first and second delay periods were prolonged by extending to 0.7–2.3 s and to 0.3–0.7 s, respectively. After each rat was trained at a training chamber over 19 sessions regardless of task performances, animal was afterwards acclimatized to another task chamber, where electrophysiological recording experiments were conducted, over two more training sessions.

### Electrophysiological Recordings

**Figure 2:**
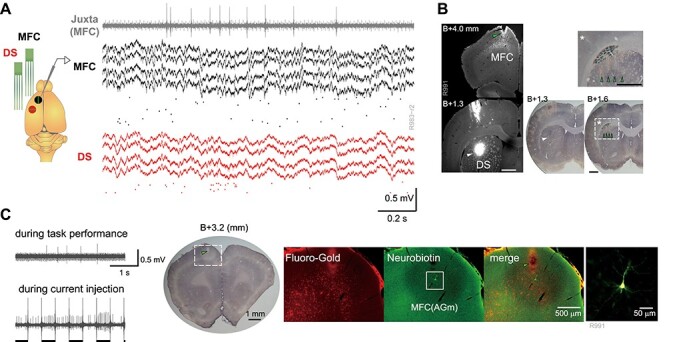
Simultaneous recording from MFC and DS. (*A*) *left*: A schematic illustration of recordings. One silicon probe (black) and a glass pipette electrode (gray) were inserted into MFC and another silicon probe into DS (red). Green dots indicate insertion position of each shank of the silicon probe. *Right*: Multiunit activity recorded with tetrodes in MFC and DS and juxtacellularly recorded activity (gray). Spikes of isolated units are depicted below multiunit signals. (*B*) *Left*: Fluorescent images show corticostriatal projection neurons labeled with Fluoro-Gold in MFC (*top*) and the injection site (white arrowhead) of Fluoro-Gold in DS (*bottom*). *Right*: Nissl stained brain sections specified by the AP coordinates from Bregma. One shown at *bottom left* is identical to the lower fluorescent image and one at *bottom right* indicates silicon probe tracks (scars, green arrowhead) in DS. The area surrounded by a dash-lined box was magnified (*top*). Scale bars show 1 mm. (*C*) *left*: Juxtacellularly recorded activity of a neuron during task performance (*top*) and current injection (*bottom*). Horizontal bars indicate epochs of periodic current injection whereas long vertical lines are artifacts by the on/off cycles of injection. *Middle*: A Nissl stained brain section including probe track (scar, green arrowhead) in MFC (agranular medial cortex, AGm). *right*: Fluorescent images reveal Fluoro-Gold labeled corticostriatal neurons and a Neurobiotin-labeled neuron in MFC. The rightmost image enlarges the inside of the box area.

After 21 training sessions were completed, two daily recording experiments were conducted for each animal (*N* = 34 rats). Multi-neuron activity was simultaneously recorded from MFC and DS of left hemisphere with two 32-channels silicon probes consisting of four shanks (0.4 mm shank separation), on which tetrode-like electrode sites were spaced vertically by 0.5 mm (A4x2-tet-7/5 mm-500-400-312, NeuroNexus Technologies, Michigan, USA). Each probe was connected to a custom-made headstage installed on either one of two fine micromanipulators (1760–61; David Kopf Instruments, California, USA) mounted on a stereotaxic frame (SR-8 N, Narishige, Tokyo, Japan). A silicon probe was penetrated vertically (depth from pia mater: 1.2 mm) into MFC, specifically into AGm (at the center of probe: +3.0–3.6 mm to Bregma, 1.0–1.4 mm to midline), and the shanks were aligned along midline (Fig. 2*A*). Another silicon probe angled posteriorly by 6° was inserted into DS through a cranial window (at the center of probe: +0.6–1.0 mm to Bregma, 2.7–3.1 mm to midline, 4.0 mm traveling distance), and the shanks were aligned along coronal suture (Fig. 2*A*). Multiunit signals were amplified by the headstages before being fed into main amplifiers (2000 gain) (Nihon Kohden, Tokyo, Japan) with a band-pass filter (0.5 Hz to 10 kHz). At the second recording session apart from muscimol injection experiments (see next section), juxtacellular activity was recorded from the MFC of left hemisphere. A glass microelectrode was prepared by a laser puller (P-2000; Sutter Instrument, California, USA) and filled with 2–3% Neurobiotin (Vector Laboratories, California, USA) dissolved in 0.5 M potassium chloride (9–19 MΩ). The electrode was inserted into MFC through the cranial hole for MFC silicon probe with a stereotaxic hydraulic micromanipulator (SM-25C, Narishige, Tokyo, Japan). Juxtacellular activity was amplified (final gain 1000) with two amplifiers (IR-283, Cygnus Technologies, North Carolina, USA; EX4–400, Dagan, Minneapolis, USA) and filtered (0.3–10 kHz). All neural data was sampled at 20 kHz with two hard-disc recorders (LX-120, TEAC, Tokyo, Japan), together with time of task events and licking each spout (left and right). After juxtacellular recording, we tried to electroporate Neurobiotin into the recorded cell with positive current injection (2–14 nA, duration of 0.5 s at 1 Hz interval, for 5–15 min) in order to visualize the recorded cell *post hoc* and to verify the recording location.

### Inactivation of the MFC

Of 34 rats, 20 reached a performance criterion (>75% reward acquisition probability) at the first and/or second recording sessions. Fifteen out of the 20 rats reached the criterion at the first recording session. For all of 15 rats, the simultaneous recording from MFC and DS was conducted at the first recording session (Day 1). Of the 15 rats, 10 and 5 were tested without and with injection of GABAa agonist muscimol into bilateral MFC at the second recording session (Day 2), respectively. For the muscimol group, the rats were placed in the body restrainer and their heads were fixed on the stereotaxic frame. A microinjection syringe (Hamilton company, Nevada, USA) filled with 0.1% muscimol (muscimol hydrobromide, Sigma-Aldrich) dissolved in 0.1 M phosphate-buffered saline (Narayanan et al. 2006) was set on a microsyringe pump (Legato 130, KD Scientific, Massachusetts, USA) which was installed on the micromanipulator (1760–61; David Kopf Instruments, California, USA). An injection needle (25 or 31 gauge) was vertically inserted into the MFC (+3.0 mm to Bregma, 1.0 mm to midline, −1.5 mm from pia mater). Muscimol solution was injected via the microsyringe pump at 0.2 μL/min by totally 0.5 μL in each hemisphere. After injections were completed, the needle was left for 5 minutes to allow for diffusion and then slowly retracted. Then, a silicon probe was inserted into DS of left hemisphere as described above. We did not record both MFC multiunit and juxtacellular activity in this case. Then, the rats were tested the behavioral task 1 hour after muscimol infusion. For control group, multiunit recording in MFC and DS and juxtacellular recording were conducted at the second recording session (Day 2).

### Histology

Animals were deeply anesthetized with Urethane (2–3 g/kg, i.p.) and then perfused intracardially with 0.9% chilled saline followed by 4% paraformaldehyde (PFA) dissolved in 0.1 M phosphate buffer (PB). The fixed brain was stored in 4% PFA overnight and then stored in 30% sucrose solution in 0.1 M PB over 2 weeks. Postfixed brains were frozen and coronally sliced into 50-μm-thick serial sections with a microtome Cryostat (HM500OM, Microm, Walldorf, Germany). The brain sections were stored in 0.1 M PB at 4°C overnight. The brain sections were subject for immunostaining to detect Fluoro-Gold and Neurobiotin. For fluorescent visualizations of Fluoro-Gold labeled neurons and Neurobiotin-loaded neuron, the brain sections were incubated with a rabbit antibody of Fluoro-Gold (AB153, 1:3000 dilution, Millipore) at 4°C overnight, followed by incubation with a goat anti-rabbit antibody of Fluoro-Gold conjugated with Alexa-594 (A11012, 1:500 dilution, Invitrogen) together with antibody Strept Avidine Alexa-488 (A11012, 1:250 dilution, Invitrogen) of Neurobiotin over 2 hours. The slices were washed with PB and mount on a slide glass and dried in a shaded box overnight. Fluorescent images were imaged with a fluorescence microscopy (AX70, Olympus, Tokyo, Japan) in order to check if Fluoro-Gold labeled neurons were observed around the silicon probe recording locations in MFC and near the Fluoro-Gold injection site in DS as well as if the Neurobiotin-loaded neurons were observed in MFC. After the fluorescent imaging, the brain sections were re-stained the Neurobiotin-loaded neuron using the avidin-biotin-horseradish peroxidase complex (Vectastain Elite ABC; Vector Laboratories, 1:200 dilution) with diaminobenzidine and nickel as demonstrated previously (Isomura et al. 2009, 2013). Finally, the slices were counterstained with Neural Red Nissl, and observed with a microscopy to check the tracks (scars) due to probe insertion. We judged the recording locations in MFC and DS and the AP coordinate of brain sections according to the rat brain atlas (Paxinos and Watson 2009).

### Data Analysis

All of behavioral and neuronal data were analyzed by custom-written MATLAB scripts (MathWorks). Of 34 rats, its reward acquisition probability of 20 rats reached the performance criterion (>75%) at the first and/or second recording sessions. We analyzed behavioral data at the time of reaching the criterion (30 recording sessions, 20 rats).

#### Logistic Regression Analysis

We estimated effects of past outcome and past choice on current choice (repeat or switch) by the following regression model (Sul et al. 2011).

(1) \begin{equation*} \log \left(\frac{\mathit{\Pr}}{Ps}\right)=\sum_{k=1}^{10}{\beta}_{Rk}R\left(i-k\right)+\sum_{k=1}^{10}{\beta}_{Ck}\left({C}_L\left(i-k\right)-{C}_R\left(i-k\right)\right)+{\beta}_0 \end{equation*}

where *p*_r_ (or *p*_s_) is the probability of repeat choice (or switch choice) in the *i*th trial. The variables R(*i*) and C_L_(*i*) (or C_R_(*i*)) indicate presence of reward delivery (0 or 1) and left (or right) choice (0 or 1) in the *i*th trial, respectively. The coefficients β_R*k*_ and β_C*k*_ represent the effect of past rewards and choices, respectively.□β_0_ is an intercept. These coefficients were calculated at each session. We checked if the coefficient was significantly different from zero by *t*-test with Bonferroni correction (Fig. 1*D*).

#### Spike Sorting, Clustering, and Refining

Spike event from multiunit (or juxtacellular) activity was isolated by a custom-made semi-automatic spike-sorting program EToS [12 (or 5) feature dimensions for 4 (or 1) channels; high-pass filter, 300 Hz; time-resolution, 20 kHz; spike-detection interval, >0.5 ms) (Takekawa et al. 2010, 2012). The sorted spike clusters were combined, divided, and discarded manually to refine single-neuron clusters by Klusters (Hazan et al. 2006). To avoid overlapping of detection of same units recorded among spatially distinct tetrodes, we checked cross-correlations of spike times among isolated units across all of tetrodes. If there was a high correlation peak only at zero time between a pair of units, one of the units was excluded from further analyses because those spikes, which originated from the same neuron were presumably recorded through different tetrodes.

For comparison of neural activity between MFC and DS, we analyzed behavioral and electrophysiological data (isolated cells from multi-neuron activity) in 12 sessions from 10 out of the 20 rats: experiment-id (reward acquisition probability, the number of well isolated cells), R982-r1 (*P* = 0.828, MFC/DS = 45/43), R983-r1 (*P* = 0.837, MFC/DS = 35/55), R983-r2 (*P* = 0.834, MFC/DS = 58/48), R985- r1 (*P* = 0.853, MFC/DS = 30/60), R985-r2 (*P* = 0.867, MFC/DS = 66/26), R986-r1 (*P* = 0.813, MFC/DS = 16/20), R991-r1 (*P* = 0.806, MFC/DS = 46/31), R1000-r1 (*P* = 0.784, MFC/DS = 12/32), R1004-r1 (*P* = 0.792, MFC/DS = 20/51), R1005-r1 (*P* = 0.794, MFC/DS = 65/64), R1009-r1 (*P* = 0.774, MFC/DS = 35/33), R1012-r1 (*P* = 0.755, MFC/DS = 40/26).

For comparison of neural activity of DS cells between Day 1 (no injection) and Day 2 (muscimol injection) in the muscimol group, we acquired DS neuronal data in Day 1 and Day 2 sessions from 3 rats and 4 rats in muscimol group, respectively. To examine effects of muscimol injection in MFC on neuronal activation of DS cells, we analyzed data from 130 and 77 DS cells in Day 1 and Day 2 sessions, respectively.

#### Event-Related Neuronal Activity

**Figure 3:**
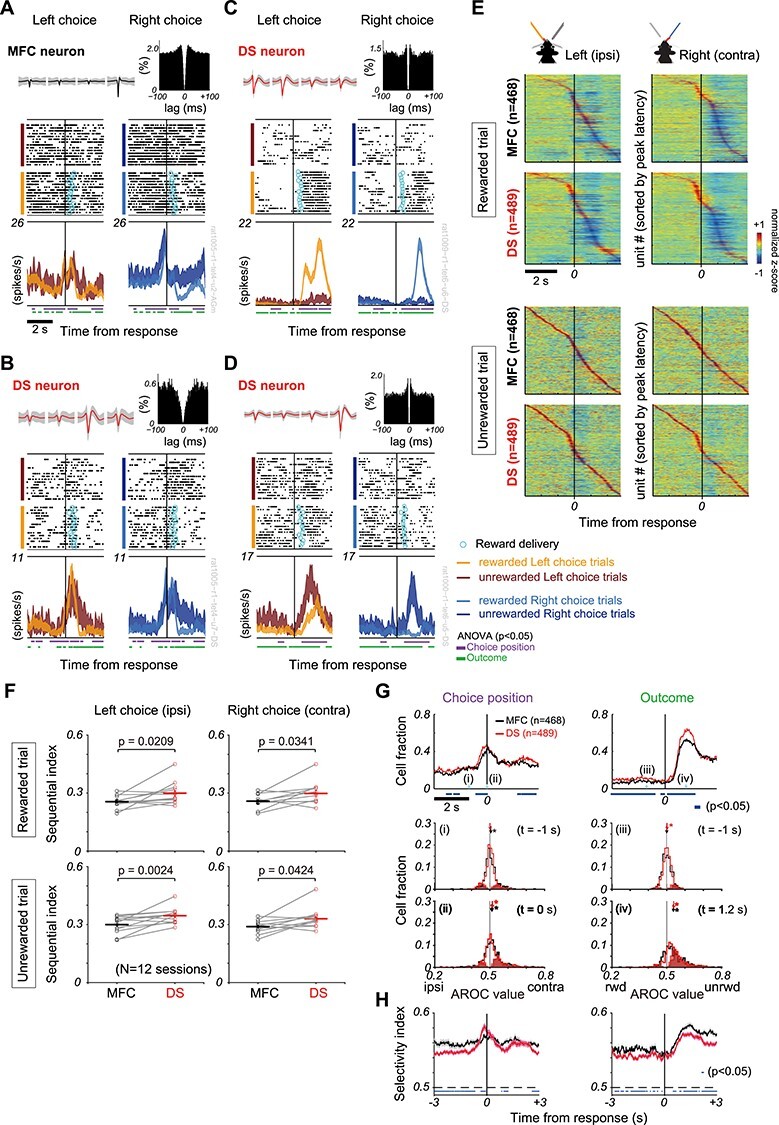
Choice and outcome coding in single neurons of MFC and DS. Below, neuronal activities are aligned to choice response (vertical line). (*A*-*D*) Representative firing patterns modulated by choices and outcomes on MFC and DS single neurons. Simultaneously recorded Right-choice selective responses of (*A*) MFC and (*B*) DS cells. These cells decreased firing rate immediately after reward delivery (cyan circle). Another DS cell increased firing rate during post-outcome periods in (*C*) rewarded and (*D*) unrewarded trials. Top row shows spike waveforms (mean ± SD) in four channels of a tetrode and auto-correlogram. Quite smaller number of unrewarded trials compared with rewarded trials. For the purpose of comparison between rewarded and unrewarded conditions, the raster plot is presented in equal number of trials because the number of unrewarded trials was much smaller than that of rewarded trials. Peri-event time histogram (PETH) shows mean ± s.e.m. of firing rate. Horizontal bars indicate the times at which firing rate in a sliding time window (200 ms, increment = 20 ms) was significantly different (two-way ANOVA, *P* < 0.05) with respect to choice (purple) or outcome (green) factor. (*E*) Normalized PETHs of MFC (*n* = 468) and DS (*n* = 489) neurons are shown in rewarded and unrewarded trials. In each condition, units are sorted by the latency of maximum absolute peaks. (*F*) Comparison of sequential index between MFC (black) and DS (red) across 12 recording sessions. Each symbol indicates the sequential index in individual recording sessions. Statistical significance was assessed by Wilcoxon signed-rank test. (*G*) *top*: Time series of the proportion of MFC (black) and DS (red) cells showing (*left*) choice or (*right*) outcome-selective activity aligned at the response time (two-way ANOVA, *P* < 0.05). Blue bars represent the times at which the cell proportions were significantly different between MFC and DS (χ2 test, *P* < 0.05). Light blue arrows with Roman numbers indicate the times at which AROC values were calculated. *Middle and bottom*: Proportion of neurons is shown against AROC values regarding their preference between contralateral and ipsilateral choices at 1 s before the response (*middle left,* i) and at the response time (*bottom left,* ii) and the preference between unrewarded and rewarded outcomes at 1 s before (*middle right,* iii) and 1.2 s after the response (*bottom right,* iv). Arrows indicate the mean AROC value and asterisk indicates that the mean value was significantly different from 0.5 (*t*-test, *P* < 0.05). Filled and opened bars show significant cells (two-way ANOVA, *P* < 0.05) and non-significant cells at the time, respectively. (*H*) Comparison of selectivity index for choice-selective neurons (*left*) and outcome-selective neurons (*right*) between MFC and DS. Thick solid lines and shaded area represent mean and s.e.m., respectively. Blue bars represent the times at which the selectivity indices were significantly different between MFC and DS (*t*-test, *P* < 0.05).

Task-event-related neuronal activity was examined on well isolated units. To make peri-event time histograms (PETHs), we calculated mean and standard error of the mean (s.e.m.) of instantaneous firing rate in a 20-ms bin around task events (Start cue onset, Go tone onset, Choice response, and Reward delivery) and the PETHs were smoothed with a Gauss filter (SD = 40 ms). To compare firing pattern across neurons and recording sessions, we transformed the PETHs into *z*-scores by using the mean and SD of control activity, which was obtained from firing rates in randomly chosen 1000 time windows of width 200 ms during the entire task period of each recording session. Then, the *z*-scores were normalized by the absolute peak value (Fig. 3*E*).

#### Neuronal Selectivity for Choice and Outcome

Statistical significance of neuronal modulation by choice and outcome was analyzed by a two-way ANOVA with choice (left and right) and outcome (reward and no-reward) factors (*P* < 0.05) for each firing rate in 200-ms wide sliding window by 20 ms increment around choice response (±3 s) (Fig. 3*G*). To quantify the preference of selectivity for choice and outcome, we conducted receiver-operating characteristic (ROC) analysis (Britten et al. 1992) for all neurons at two specific times concerning choice selectivity (1 s before the response as a baseline and at the time of response when the fraction of choice-selective neurons was maximal) and outcome (1 s before and 1.2 s after the response time, where the fraction of outcome-selective neurons reached a maximum at the latter). The proportion of trials for a condition (left choice or rewarded trials) was plotted against that of trials for another condition, (right choice or unrewarded) while changing a criterion for the firing rate. If the area under the plotted ROC curve (AROC) was 0.5 (0 or 1), the two conditions are indistinguishable (or completely different, respectively). If AROC was larger (smaller) than 0.5, firing rate is larger in right choice (left choice) or unrewarded outcome (rewarded outcome) than under the opposite condition. To compare neural selectivity for choice and outcome between MFC and DS, the AROCs of neurons showing statistical significance by two-way ANOVA were transformed to a selectivity index as follows: Selectivity index = |AROC—0.5| + 0.5 (Fig. 3*H*). To investigate the neural correlate of task performance in MFC inactivation experiments, this selectivity index was calculated. For this analysis, AROCs for choice and outcome were calculated in 1-s windows immediately after a response and 0.5 s after a response, respectively (Fig. 7*F* and *G*).

#### Sequential Index

For quantitative comparison of neural dynamic between MFC and DS, we calculated sequential index which was developed previously (Zhou et al. 2020). The normalized sequential index was separately calculated in four conditions based on two choice positions and two outcomes:

(2) \begin{equation*} PE=\frac{-\sum_{k=1}^B{p}_k\log \left({p}_k\right)}{\log (B)} \end{equation*}

(3) \begin{equation*} TS=1+\left\langle -\sum_{i=1}^N{R}_{it}\log \left({R}_{it}\right)/\log (N)\right\rangle_t \end{equation*}

(4) \begin{equation*} Sequential\ index=\sqrt{PE\ast TS} \end{equation*}

where PE and TS indicate peak entropy and temporal sparsity, respectively. The peak entropy provides the entropy of distribution of peak activity times over the whole neural population in each recording session. B is the number of bins (bin = 50 ms) for estimating the peak time distribution (120 bins) and *N* is the number of units; ${p}_k$ refers to the number of units with peaks in time bin *k* normalized by *N*; *R*_it_ represents the mean firing rate of unit *i* in time bin *t* and is normalized by the sum of the mean firing rates of all units at time *t*; < >_t_ denotes the time average. The temporal sparsity provides a measure of entropy of the distribution of normalized activity in any given bin. TS is maximized if a single unit accounts for all the activity in each time bin. The Sequential index approaches unity when the peak times of individual units homogeneously tile the entire duration and only one unit is active at every moment without overlaps between their temporal fields.

#### Analysis of Neural Trajectory Separation

All analyses for population activity were separately performed on data sampled at individual recording session (*N* = 12 sessions). Fisher’s linear discriminant (Bishop 2006) was used to find the degree of discrimination between neural trajectories classified into two conditions (e.g., Left and Right choices). To define a Ch-hyperplane, we constructed the distributions of the corresponding ***r***(*t*), which is *N*-dimensional population firing rate vector (*N* corresponds to the number of population of neurons), separately for Left and Right choice trials in each 200-ms sliding window by 50 ms increment time around choice response (±3 s). Our task is to find a hyperplane, or equivalently the normal vector **w** of this hyperplane, that best divides the two distributions projected onto the direction of **w**. If the two distributions have the means and covariance matrices **m**_1_, Σ_1_ and **m**_2_, Σ_2_, respectively, we can obtain **w** by maximizing the ratio of the between-class variance to the within-class variance,

(5) \begin{equation*} S={\left({\mathbf{w}}^T{\mathbf{m}}_1-{\mathbf{w}}^T{\mathbf{m}}_2\right)}^2/{\mathbf{w}}^T\left({\Sigma}_1+{\Sigma}_2\right)\mathbf{w}, \end{equation*}

where **w**^T^**m**_1_ and **w**^T^**m**_2_ are the means of the two projected distributions and **w**^T^Σ_1_**w** and **w**^T^Σ_2_**w** are their variances. We can show that *S* is maximized if $\mathbf{w}\propto{({\Sigma}_1+{\Sigma}_2)}^{-1}({\mathbf{m}}_2-{\mathbf{m}}_1)$. The coefficient of proportion can be determined by the normalization condition: |**w**| = 1.

Thus, we can calculate the discrimination function between Left and Right choice trials as $S={({r}_L-{r}_R)}^2/({\sigma}_L^2+{\sigma}_R^2)$ from the means (*r_L_*, *r_R_*) and standard deviations (*σ_L_*, *σ_R_*) of the trial-by-trial firing rates in these trials. The discrimination degree between the two clusters is defined as *d’* = *S*.

Out of the 12 sessions, 9 sessions (7 rats) were subject to further analyses of neural trajectory as those data reached our criterion (cell number ≧25 in both regions per session) in order to keep a sufficient number of cells (Fig. S3). The ***r***(*t*) in each 0.2-s wide window (0.2 s increment time) around choice response (±3 s) was projected onto the axis orthogonal to the hyperplane by calculating inner product of ***r***(t) and the normal vector **w**_choice_**,** which yielded maximal degree of discrimination (*d’*). For instance, population rate at time *t* vectors was projected onto the axis orthogonal to “Ch-hyperplane,” *v_t_* = < ***r***(*t*), ***w***_choice_>. In this case, we termed the projected rate vector “Ch-projected trajectory.” We performed the same procedure to find “Ot-hyperplane” (**w**_outcome_) by classifying trials into rewarded and unrewarded conditions, and to obtain the projected rate vector “Ot-projected trajectory.”

**Figure 4:**
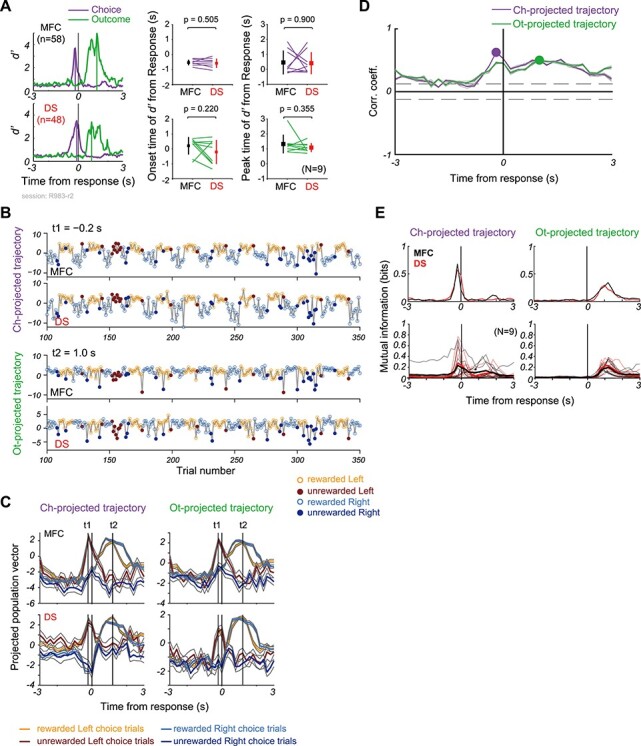
Temporal correlations of neural trajectories between MFC and DS. Unless otherwise stated, all results are shown for a typical session (R983-r2). (*A*) *left*: Time series of discrimination degree (*d’*) for neural populations simultaneously recorded in MFC (*n* = 58, *top*) and DS (*n* = 48, *bottom*). Vertical lines indicate the peak times of *d’* measured between different choices (purple) and outcomes (green). *middle*/*right*: Comparison of the onset times (*middle*) and peak times (*right*) of *d’* between MFC and DS (*N* = 9 sessions) at Ch- and Ot- hyperplanes. Squares and error bars denote mean and SD. P-value was assessed by paired *t*-test. (*B*) Trial-based distances of choice- and outcome-projected trajectories from the separating hyperplanes at t1 (−0.2 s from response time, *top*) and t2 (1.0 s after response time, *bottom*) are shown in MFC (*1st row*) and DS (*2nd* row) in this session. (*C*) Trial-averaged (*left*) choice-projected and (*right*) outcome-projected trajectories of population vectors in MFC (*top*) and DS (*bottom*) in the session. Gray lines show standard errors. Vertical lines indicate times t1 (−0.2 s) and t2 (+1.0 s), as shown in B, at which the trajectories were near maximally separated on choice and outcome axes, respectively. (*D*) Time evolution of Pearson’s correlation coefficients was calculated between MFC and DS projected trajectories shown in B. Purple and green lines represent the mean correlation coefficients, and gray solid lines show individual correlation coefficient calculated from randomly sampled 431 trials. Circles indicate the times of the maximum correlation. Horizontal gray dashed lines represent a criterion for statistical significance (*P* = 0.01, *r* = 0.124, *t* = 2.588, df = 430). (*E*) *top*: Mutual information about choice (*left*) and outcome (*right*) obtained from the trajectories in MFC (black) and DS (red) shown in B. *bottom*: Mutual information from 9 sessions (thin) and the average (thick). Asterisk indicates statistical significance (Mann–Whitney U test, *P* < 0.05).

To determine the onset time of increases in the degree of discrimination between neural trajectories, we calculated the mean and SD of the degree of discrimination in the period ranging from −3 to −1 s from response time as a baseline level. We determined the first bin at which the degree of discrimination exceeded the mean plus 3SD as the onset time (Fig. 4*A*).

#### Mutual Information

To quantify the difference in Ch-projected trajectory (or Ot-projected trajectory), we calculated the mutual information *I* (Shannon 1948) between choices (or outcomes) and the projected population rate vectors *v_t_*:

(6) \begin{equation*} I(C;{V}_t)=H(C)-H(C|{V}_t)=-{\sum}_cp(c)\log p(c)+\langle{\sum}_cp(c|{v}_t)\log p(c|{v}_t)\rangle \mathrm{v},\end{equation*}

where *H* denotes entropy, *C* and *V_t_* denote the set of choices (or outcome) *c* and the set of projected population activity *v_t_*, respectively, and *p*(*c*) is the choice probability (or outcome probability), *p*(*c*|*v_t_*) is the conditional probability of choice (or outcome) *c* when *v_t_* is observed at time *t*, and the parenthesis means an averaging over the values of *v_t_*. The mutual information was calculated at each time window (bin = 0.2 s) around choice response (±3 s) as demonstrated before (Handa et al. 2017).

#### Pairwise Correlation Analysis of Population Activity

**Figure 5:**
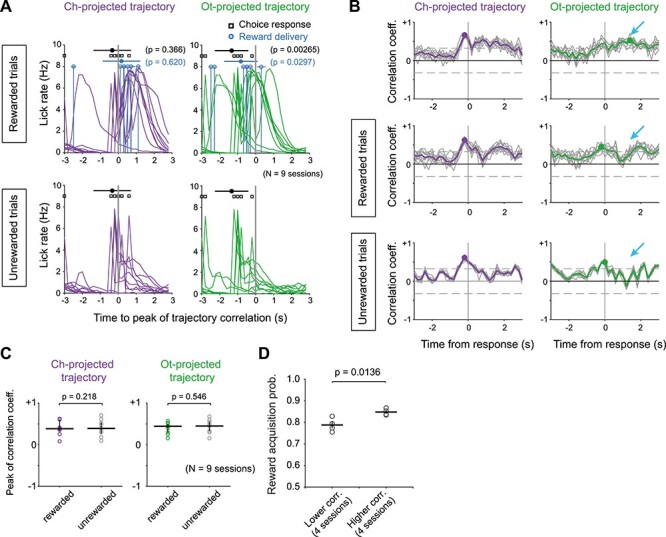
Behavioral variable when the correlation of trajectories reached peak. (*A*) Session-by-session licking rate, choice response (black) and reward delivery (light blue) are aligned at the time when Ch-projected (purple) and Ot-projected (green) trajectories showed peak correlations between MFC and DS. The origin of time refers to the time of peak correlations. Means (filled symbols) and SDs (horizontal lines) of choice response and reward delivery time are shown (*N* = 9 sessions). (*B*) Time evolution of trajectory correlations in rewarded (*middle*), unrewarded (*bottom*), and mixed (*top*) trials for a rat shown in Figure 4C. Arrows indicate the times of peak correlation in the mixed trial condition. We randomly selected 63 trials in each condition and calculated correlation coefficient (gray solid line). We repeated this procedure by 10 times. Averaged correlation is represented by colored thick line. Circles indicate the peak values and times. Horizontal gray dashed lines represent a criterion for statistical significance (*P* = 0.01, *r* = 0.3223, *t* = 2.659, df = 62). (*C*) Comparison of peak correlation coefficients between rewarded and unrewarded trials. (*D*) Reward acquisition probability between 4 sessions with lower trajectory correlations and 4 sessions with higher trajectory correlations. Thick and thin bars represent the mean and s.e.m., respectively.

A Pearson correlation was performed for Ch-projected trajectory and Ot-projected trajectory between MFC and DS at each time window (bin = 0.2 s) around choice response (±3 s). Because the correlations are sensitive to the number of trials, we randomly selected 431 trials, which was the minimum total number of trials among 9 sessions, and calculated correlation coefficient so that the correlation coefficient was calculated with the same total number of trials sampled. We repeated this procedure 10 times to obtain mean correlation coefficients (Figs 4*D* and S5). To compare the correlations of trajectories between rewarded and unrewarded trials, we calculated the correlation coefficients for randomly selected 63 trials, which was the minimum total number of unrewarded trials among 9 sessions, for each outcome condition (only rewarded trials, only unrewarded trials, trials without distinction). We repeated this procedure 10 times to obtain mean correlation coefficients (Fig. 5*B*). Statistical significance of correlation was assessed by transforming correlation coefficient into t-value and comparing with t-distribution (*P* = 0.01).

#### Partial Correlation

To examine if the correlations of neural trajectories between MFC and DS were observed by another task event as a confounder, we calculated partial correlation coefficient by taking account of possible influences from the last outcome event (rewarded or non-rewarded). The partial correlation R_xy-z_ was calculated in terms of pairwise Pearson’s correlations (randomly sampled 431 trials, 10 repetitions) as follows:

(7) \begin{equation*} {R}_{xy-z}=\frac{\left({R}_{xy}-{R}_{xz}\ {R}_{yz}\right)}{\sqrt{\left(1-{R_{xz}}^2\right)\left(1-{R_{yz}}^2\right)}} \end{equation*}

Here, *R_xy_* reveals correlation coefficient between MFC and DS neural trajectories calculated above, whereas *R_xz_* and *R_yz_* reveal the correlation coefficients between MFC and the last outcome (rewarded = 1 or unrewarded = −1) and that between DS and the last outcome, respectively. We repeated this procedure and averaged the partial correlation to get peak correlations (Fig. S4). If the last outcome affected the correlation between the trajectories, i.e., if either ${R}_{xz}$ or ${R}_{yz}$ is not zero, the partial correlation (*R_xy-z_*) should be different from trajectory correlation (*R_xy_*).

#### Cross-Correlation Analysis of Spike Times

**Figure 6:**
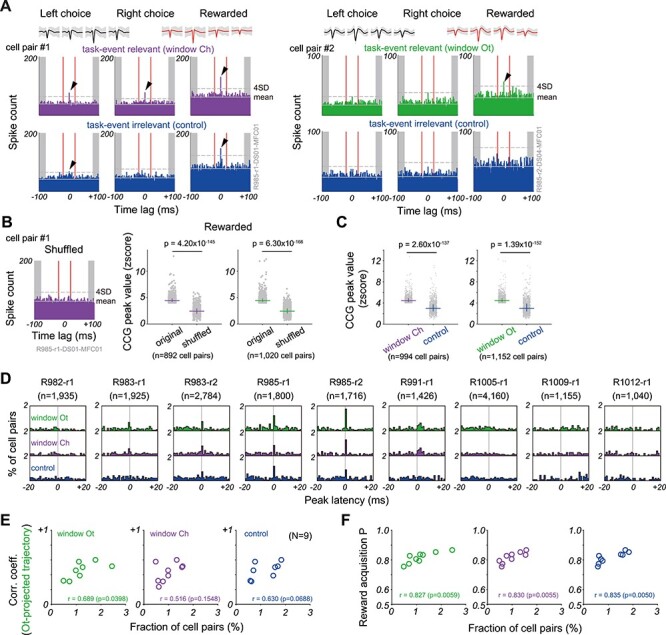
Relationships among MFC-DS spike synchrony, trajectory correlation and task performance. (*A*) Spike CCGs of two MFC-DS cell pairs (left: cell-pair#1, right: cell-pair#2). For each cell pair, time lags of MFC cell spike firing relative to DS cell spike firing were calculated in task-event relevant (*middle row*) and irrelevant (*bottom row*) time windows. Average and SD (gray shade) of spike waveforms recorded with tetrodes in MFC (black) and DS (red) are also shown (*top row*). *left and middle columns*: Left or Right choice trials with arbitrary outcomes, respectively. *Right column*: rewarded trials with arbitrary choice positions. Windows Ch (purple) and Ot (green) refer to the analyzed time windows centered around the peak MFC-DS correlations of Ch-projected and Ot-projected trajectories, respectively. Task-event irrelevant CCG gives a control and was calculated at a randomly selected time point. Arrowhead indicates the peak of CCGs above the mean + 4SD of the baseline defined by spike counts from −100 to −80 ms and from +80 to +100 ms (gray shaded ranges). (*B*) *left*: CCGs of the cell pair #1 were calculated in the shuffled trial orders in rewarded trials. *Right*: Normalized CCG peak amplitudes were calculated for all cell pairs showing significant CCG peaks in windows Ch (purple) and Ot (green). The peak amplitude is compared with those obtained in shuffled trial orders. Horizontal lines and error bars show the median and 1^st^/3^rd^ quartiles, respectively. P-values were calculated by Wilcoxon signed-rank test. (*C*) CCG peak values of cell pairs in windows Ch (purple) and Ot (green) are compared with those in task-event irrelevant windows (blue). Each dot represents a cell pair with a significant CCG peak (peak >4SD). Horizontal lines and error bars show the median and 1^st^/3^rd^ quartiles, respectively. P-values were calculated by Wilcoxon signed-rank test. (*D*) Proportion of MFC-DS cell pairs that displayed an excess amount of spike coincidences in rewarded trials was plotted for the 9 sessions against CCG peak time lags. The numbers of such cell pairs are given in parentheses. (*E*) Correlation coefficients between MFC and DS Ot-projected trajectories are plotted against the fraction of MFC-DS cell pairs showing a CCG peak within ±2 ms for different time windows. (*F*) Relationship between such MFC-DS cell pairs and animal’s reward acquisition probability. Symbols represent individual sessions, and Pearson correlation coefficient and P-value are denoted for each diagram.

For the 9 datasets which were subject to above neural trajectory analyses, we calculated cross-correlograms (CCGs) under 4 conditions (left choice trials, right choice trials, rewarded trials, and unrewarded trials) for all MFC-DS cell-pairs. We used spike times in a 5-s wide window centered on a specific time at which a maximum absolute correlation of neural trajectories was yielded at Ch-hyperplane (or Ot-hyperplane) referred as window Ch (or window Ot) (Figs 4*D* and S5). As a control, we calculated task-event irrelevant CCGs by randomly sampling X time points within the same recording session for the same cell-pair. X corresponds to the number of trials used for calculation of task-event relevant CCGs under each condition. For example, the CCG between MFC and DS cells was calculated by counting spikes of MFC cell in a window ranging from −0.1 to +0.1 s relative to each spike time of DS cell (bin = 1 ms). Spike synchrony was detected by comparing the peak value in CCGs within ±20 ms with the mean + 4SD of values in a baseline window (−100 to −80 ms and + 80 to +100 ms) under each condition. If the peak value was larger than this criterion, we judged that the neuron pair showed spike synchrony. We did not assess spike synchrony if the mean of its baseline values was less than 1 due to scarce spiking. We did not look into negative spike correlation. For each cell-pair showing significant CCG peak values, we also calculated CCGs by shuffling trials between MFC and DS cells (Fig. 6*B*). To compare peak value of CCGs, each CCG was z-scored by the mean and SD of the baseline values (Fig. 6*B*, *C*).

## Results

### Adaptive Outcome-Based Behavior Under Head-Restrained Condition

Behavioral data were analyzed for 20 rats that reached the criterion (reward acquisition probability >75%, see section Materials and Methods) during electrophysiological recordings. Head-restrained rats were trained to perform an outcome-based two-choice task in which choices were made by licking one of two spouts (Left and Right, Fig. 1*A*). In each block of trials only one spout delivered reward, and the rewarding spout was systematically switched without any sensory feedback when the accumulated number of rewarded trials exceeded 10 in each block. The rats figured out the reversal of choice-reward contingency within subsequent several trials by monitoring no-reward events, time-out, and reward acquisition (Fig. 1*B*). The rats tended to select the same spout as chosen in the last rewarded trial, but they switched the choice following one to several unrewarded trials (Fig. 1*B*, *C*). Whether the choice pattern was biased by previous choice positions (Left and Right) or previous outcomes (reward or no-reward) was further examined by using logistic regression (Fig. 1*D*, see Materials and Methods). We found that the last outcome was most influential on the current choice type (repeat or switch) regardless of the previous choice positions (*t*-test with Bonferroni correction, *P* = 3.73 × 10^−15^) and that the last choice position most strongly affected the current choice position regardless of the previous outcomes (*t*-test with Bonferroni correction, *P* = 5.17 × 10^−6^). Thus, the choice pattern of the rats resembled the so-called “win-stay” and “lose-shift,” which is indeed the optimal strategy to maximize reward in the present task.

Trial-by-trial licking behavior was also modulated by outcome events. Rats generally licked one of the two spouts immediately after the appearance of Go tone in both Left and Right choice trials. Then, after reward delivery, they showed fast and rhythmic licking at around 6 Hz. In contrast, in unrewarded trials the rats showed sparse licking at around 1 Hz during outcome period (Fig. 1*E*). The lick rate of population data was significantly different before and after reward delivery in rewarded trials (Fig. 1*F*, Wilcoxon signed-rank test, *P* = 0.00295) and between rewarded and unrewarded outcome periods (Wilcoxon signed-rank test, *P* = 1.50 × 10^−6^).

### Simultaneous Multi-Neuron Recordings from MFC and DS

To test whether and how neuronal population activities of the MFC-DS circuit are collectively coordinated during the outcome-based action selection, we recorded multi-neuron activity in the MFC and DS of the left hemisphere using two silicon probes. In addition, we simultaneously performed juxtacellular recordings of Neurobiotin-loaded neurons near the silicon probe inserted into the MFC (Fig. 2*A*). It is known that MFC, particularly the rostral agranular medial cortex (AGm), is one of the main regions projecting excitatory inputs to DS (Wilson 1987; Cheatwood et al. 2003; Reep et al. 2003). To confirm projections from the recording site in the MFC to that in the DS, we injected a retrograde tracer Fluoro-Gold into the DS (see section Materials and Methods). Fluoro-Gold-labeled corticostriatal neurons were primarily observed in the layers 3 and 5 of the AGm together with the track of a probe for MFC recording. In some case, Neurobiotin-loaded neuron was observed within the area containing Fluoro-Gold labeled neurons (Fig. 2*B*, *C*). The track of a probe for DS recording could also be identified at or near the injection site in DS (Fig. 2*B*). These results confirmed that multi-neuron activity was likely recorded from the synaptically connected subregions of the MFC and DS.

### Single MFC and DS Cells Encode Both Positive and Negative Outcomes

We analyzed the event-related firing patterns of well-isolated single neurons recorded simultaneously from MFC (*n* = 468, mean ± SD = 39 ± 18 cells per session) and DS (*n* = 489, mean ± SD = 40 ± 14 cells per session could obtain sufficiently) in 10 rats that yielded sufficiently many neurons for the analysis. The recordings were performed in the left hemisphere while these rats were performing 12 recording sessions of the learned task (Materials and Methods). The firing rates of these MFC and DS cells were correlated with either choices (Left vs. Right) or outcomes (Reward vs. No-reward) (two-way ANOVA, *P* < 0.05, see section Materials and Methods). Many outcome-modulated neurons decreased firing rate during the period of positive outcome (Fig. 3*A*, *B*). For positive outcome, only a minority of neurons increased firing rate (Fig. 3*C*). The reward-induced activity suppression in the majority of MFC and DS neurons is somewhat unexpected. In contrast, the majority of neurons did not raise firing rate for negative outcome. However, in some neurons firing rate was increased most strongly for negative outcome (Fig. 3*D*).

Peri-event time histograms (PETHs) also indicated a prominent decrease in firing rate in the majority of MFC and DS cells after reward delivery (Fig. 3*E*). Interestingly, the PETHs revealed highly parallel sequential dynamics (neural trajectories) of MFC and DS cells (Figs 3*E* and S1). In unrewarded choice trials, the PETHs were noisy due to the small number of trials. In particular, the trajectory in MFC is close to a straight line for unrewarded Right choices and hence may represent a mere artifact of sorting noise fluctuations. However, as shown below, the DS ensemble downstream to MFC exhibits clear sequentiality, suggesting that the trajectory in MFC had some impact on the sequential dynamics in DS. We compared the extent to which neural dynamics can be regarded as sequential between MFC and DS by means of sequential index, which was previously developed for a similar comparison between secondary motor cortex and dorsal striatum (Zhou et al. 2020) (see section Materials and Methods). The sequential index was significantly higher in DS than in MFC (Fig. 3*F*) (Wilcoxon signed-rank test, *P* < 0.05), suggesting that neural dynamics were more sequential; i.e., individual neurons have more localized temporal individual neurons have more temporally localized activation patterns, in DS than in MFC.

The fractions of choice- and outcome-modulated neurons also evolved similarly in the MFC and DS except that the fraction was greater in the DS than in the MFC for a large portion of task period (Fig. 3*G*). To quantify the degree of preferences for choice and outcome selectivity, we conducted receiver operating characteristic (ROC) analysis of firing rates and calculated the area under ROC curve (AROC) (see section Materials and Methods). AROC is 0.5 in the absence of bias but takes a value of 0 or 1 when neuronal responses are completely biased towards either side (Left vs. Right, rewarded vs. unrewarded). In both MFC and DS, choice-selective neurons exhibited a bias towards Right choice (i.e., the contralateral side) responses at the response time (Fig. 3*G*-ii), and outcome-selective neurons were biased towards unrewarded outcomes at 1.2 s after the response time at which the fraction of selective neurons reached a maximum (Fig. 3*G*-iv). At the group level, both choice- and outcome-selective neurons showed significantly higher selectivity in the MFC than in the DS for a large portion of task period (Fig. 3*H*, *t*-test, *P* < 0.05), where the selectivity index was defined as |AROC—0.5| + 0.5. Thus, choice- and outcome-modulated neurons show similar dynamical evolution in MFC and DS, but their fractions and degrees of selectivity were significantly different between the two brain regions. These results suggest parallelism between MFC and DS in the neural population coding of task-relevant information in this outcome-based choice task.

Since the rats continued to perform well in the sessions following the reversal of reward positions, we addressed if the rats showed any choice behaviors predictive of the reversal and whether MFC and DS neurons showed any activity suggesting predictive choice behaviors. The choice probability did not significantly change before and after the reversal but significantly changed before and after the first trial after the reversal (one-way ANOVA followed by Tukey–Kramer multi comparison test, *P* < 0.05) (Fig. S2*A*). In both MFC and DS, the averaged collective firing rates also did not significantly change across trials prior to the reversal (one-way ANOVA, *P* > 0.05) (Fig. S2*B*). These results suggest that the rats could not predict the reversal of reward position in advance and neural activities in MFC and DS did not show any signature of prediction.

### Functional Correlation of Neural Trajectories Between MFC and DS

The above-mentioned similarity in neural dynamics raises a question about the way neural population activities are coordinated in MFC and DS to encode information about decision making. To clarify this, we explored how the neural trajectories encoding Left and Right decisions were separated in each session (12 sessions in total) by using Fisher’s linear discriminant (Materials and Methods). This method yields a hyperplane that optimally separates datasets belonging to two categories, such as left/right choices or positive/negative outcomes, in the high dimensional space of the instantaneous population rate vectors (Bishop 2006; Handa et al. 2017; Kurikawa et al. 2018). We calculated discriminability index *d’*, which is the distance between the categorized rate vectors measured orthogonally to such a hyperplane (Fig. S3*A*). The peak values of *d’* generally depended on the dimensionality (i.e., *N*) of population vector (Pearson correlation analysis, Ch-hyperplane, *r* = 0.739, *P* = 0.0060 for MFC, *r* = 0.630, *P* = 0.0280 for DS; Ot-hyperplane, *r* = 0.616, *P* = 0.0327 for MFC, *r* = 0.612, *P* = 0.0344 for DS) (Fig. S3*B*). In particular, the peak of *d’* for Ch-hyperplane was obscure when the number of MFC neurons was smaller than 25. To keep a sufficient number of cells (*n* ≧ 25 in both regions per session), in the analyses below we omitted three data sets (R986-r1, R1000-r1, R1004-r1 in Fig. S3*A*) and only used 9 datasets (R982-r1, R983-r1, R983-r2, R985-r1, R985-r2, R991-r1, R1005-r1, R1009-r1, R1012-r1).

The value of *d’* for choice-selective or outcome-selective trajectories peaked around the time of choice response or outcome, respectively (Figs 4*A* and S3*C*). Somewhat unexpectedly, the peak times as well as the onset times were not significantly different between MFC and DS (Fig. 4*A*). Neural trajectory evolution was further studied in each session in terms of the population vector projected onto an axis orthogonal to Ch-hyperplane (Ch-projected trajectory) or Ot-hyperplane (Ot-projected trajectory). Figure 4*B* and *C* shows trial-by-trial values of the projected trajectories and trial-averaged evolution in a session, respectively. In both MFC and DS, the trajectories evolved similarly, diverging along with the choice axis (Left or Right) around the response time (t1: 0.2 s before choice response) and then along with the outcome axis (rewarded or unrewarded) during the outcome period (t2: 1 s after choice response). Interestingly, the trajectories evolving in the MFC and DS dynamically changed their relationship during the task. In contrast to that the trial-averaged Ot-projected trajectories gradually increased correlation between MFC and DS around the outcome event (*r* = 0.50), the trial-averaged Ch-projected trajectories increased correlation only transiently around the choice event (*r* = 0.62) (Fig. 4*C*, *D*), indicating a rapid information transfer between the two regions.

The observed correlations between MFC and DS neural trajectories were modest and could be a reflection of the fact that both areas were engaged in the same behavioral task. To exclude this possibility, we calculated partial correlations between the neural trajectories remaining after subtracting the influences of the last outcomes (rewarded and unrewarded) on the trajectory correlation (Materials and Methods). As shown in Figure 1*D*, behavioral choices were significantly influenced by the last outcomes. Both time courses (Fig. S4*A*: c.f. Fig. 4*D*) and peak values (Fig. S4*B*, paired *t*-test, *P* > 0.05) of the averaged partial correlation remained almost unchanged from those of the original averaged correlation. These results suggest that the trajectory correlation between MFC and DS was not simply due to their involvements in the same task event.

The choice and outcome information carried by the neural trajectories also evolved similarly in MFC and DS (Fig. 4*E*). The amount of information varied from session to session and tended to be greater in MFC than in DS. However, the mutual information averaged over nine sessions was not statistically different between MFC and DS around the time of choice response (Mann–Whitney U test, *P* > 0.05).

The coherence of the trajectories between MFC and DS varied from session to session (Fig. S5). The inter-region trajectory correlation was modestly strong (Pearson correlation coefficient was about 0.5 or greater) in 4 sessions (R983-r2 in Fig. 4, R983-r1, R985-r1, and R985-r2 in Fig. S5). In the other 5 sessions, while the Ot-projected trajectories were similar, the Ch-projected ones behaved differently in MFC and DS and the correlation between the two areas was also weaker (Fig. S5).

### Behavioral Relevance of Neural Trajectories in MFC and DS

Next, we ask the behavioral implications of highly similar evolution of neural trajectories and task-relevant information in MFC and DS. We first investigated the temporal relationships among licking behavior, choice responses, reward delivery and the times of peak trajectory correlation between MFC and DS. The licking rate quickly reached a peak when the choice response was made and then decayed during the outcome period (Fig. 5*A*). The decay was slow in rewarded trials while it was much faster in unrewarded trials. The peak correlation times of the Ch-projected trajectories tended to cluster around choice response (*t*-test, *P* = 0.366) and reward delivery (*t*-test, *P* = 0.620), while the correlation of Ot-projected trajectories exhibited a peak significantly later than these events (*t*-test, *P* = 2.65 × 10^−3^ for choice response and 0.0297 for reward delivery) and remained high during the outcome period (Fig. 5*B*). Thus, the epochs of highly correlated Ch- and Ot-projected trajectories likely occur in relation to licking behavior or the intake of reward, respectively. The trajectory correlations displayed different temporal profiles in rewarded and unrewarded trials (Fig. 5*B*), but their peak correlations were not significantly different between the two trial types (Wilcoxon signed-rank test, *P* = 0.218 for Ch-projected and 0.546 for Ot-projected trajectories) (Fig. 5*C*), suggesting that the enhanced correlations reflect the monitoring rather than the delivery of reward. The degree of trajectory correlations varied from session to session. Interestingly, the reward acquisition probability was significantly larger (*P* = 0.0136, two-sample *t*-test) in 4 sessions with higher trajectory correlations during choice and outcome periods than in 4 sessions with lower trajectory correlations (Fig. 5*D*).

To further show the behavioral relevance of neural trajectory, we examined whether the trajectory switched in either or both of MFC and DS when the rats switched their choices after unrewarded trials. In general, the rats switched their choices after a reversal of reward block within a couple of unrewarded trials (Fig. 1*B*, *C*). We hypothesized that during the unrewarded trials neural trajectory should cross the hyperplane (unless otherwise stated, “hyperplane” refers to the Ch-hyperplane) from one side to the opposite side (i.e., “Right to Left” or “Left to Right”) before a choice response in the next trial. To show this, we separately analyzed the trajectories when the rats successfully switched their choices after unrewarded trials and when they failed to do so. In 3 sessions out of 9, we observed a gradual crossing of the MFC trajectory during inter-trial interval (ITI) period when the rats switched their choices after unrewarded trials (Fig. S6*A*). By contrast, such a crossing of trajectories was not observed when the rats failed in switching behavior (Fig. S6*B*). These results suggest that decision-making on behavioral switching was processed during ITI period after unrewarded outcomes. Intriguingly, a similar crossing of trajectories was rare in the DS and observed only in one session (Fig. S6*A*). Thus, the crossing was more prominent in the MFC than in the DS although the results were not strong enough to convincingly determine which region dominated the switching of choice responses.

### Synchronous Spiking When Neural Trajectories were Highly Correlated Between MFC and DS

As shown above, neural trajectories in MFC and DS exhibit task event-dependent modulations of coherence. This result raises questions about whether neuronal firing is also temporally correlated between these regions and whether correlated spikes, if any, contributed to neural trajectories. To study these questions, we calculated cross-correlograms (CCGs) of MFC-DS cell pairs in the time window in which MFC-DS correlations of trajectories were maximized (see Figs 4*D* and S5) (Materials and Methods). Then, we compared task-event relevant and irrelevant CCGs in three trial categories (i.e., Left-choice, Right-choice, and rewarded trials).

Our analysis revealed MFC-DS cell pairs displaying statistically significant spike synchrony in a task-event-dependent manner (Fig. 6*A*). Shuffling spike trains across trials within the individual pairs eliminated the peaks in CCGs (Fig. 6*B*), implying that these cell pairs fired with a close temporal relationship in each trial. The peak values of CCGs showed significant correlations between MFC and DS (>4SD of the base line) in choice and outcome related windows, whereas these values were greatly decreased if CCGs were calculated in task-event-irrelevant windows (i.e., at random time points) (Fig. 6*A*, *C*).

Furthermore, spike synchrony tended to occur when neural trajectories were highly correlated between MFC and DS. The spike count of more MFC cells exhibited significant peaks at almost vanishing time lags from the firing of DS cells in 4 sessions (R983-r1, R983-r2, R985-r1 and R985-r2 in Fig. 6*D*) and the maximum correlation coefficients between the MFC and DS trajectories were near or over 0.5 around these peak times (Figs 4*D* and S5). By contrast, the CCG peak distributions did not show such a bias in the other 5 sessions (R982-r1, R991-r1, R1005-r1, R1009-r1, and R1012-r1) and the trajectory correlations were also weak compared to the previous 4 sessions (Fig. S5). Interestingly, the correlation coefficient between the MFC and DS Ot-projected trajectories was positively correlated with the fraction of cell pairs showing CCG peaks within ±2 ms in rewarded trials (Pearson correlation test, window Ot, *r* = 0.689, *P* = 0.0398) (Fig. 6*E*), and this fraction was in turn correlated with the probability of animal’s reward acquisition (Fig. 6*F*). These results suggest a functional relationship between the precisely timed firing and the coherent evolution of neural trajectories between MFC and DS for boosting animal’s task performance. Spike synchrony likely coordinates task-related neural population activities in the two regions. The CCGs calculated for unrewarded trials did not show significant peaks (Fig. S7). However, the small number of unrewarded trials and the sparse firing of DS neurons in such trials made it difficult to examine whether the absence of clear peaks reflected the technical limitations or biological reality (see Fig. S7). Therefore, we excluded unrewarded trials from further analyses.

### Inactivation of MFC Attenuated Neural Representation of Choice and Outcome in DS

**Figure 7:**
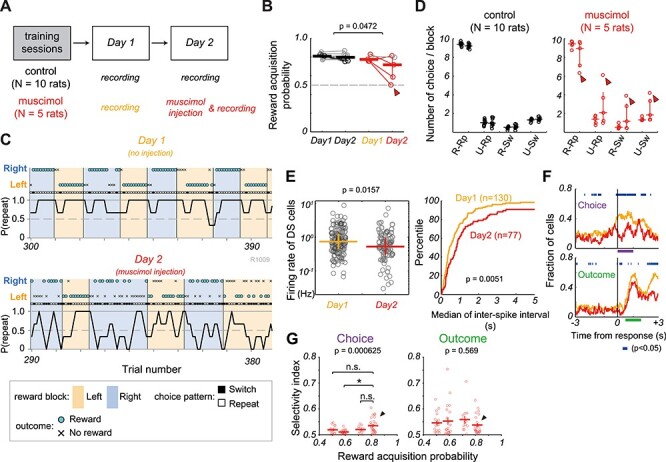
Inactivation of MFC attenuated neuronal representation of choice and outcome in DS. In statistical comparisons, horizontal and vertical lines indicate the median and 1st/3rd quartiles, respectively. (*A*) Experimental schedules for control (*N* = 10 rats) and muscimol (*N* = 5 rats) groups. At the first session (Day 1), all 15 rats underwent the recordings of multi-neuron activity from MFC and DS without any pharmacological treatments. At the second session (Day 2), we injected muscimol into the MFC of 5 rats (muscimol group) and recorded multi-neuron activity from DS. For the remaining 10 rats (control group), neural activities were recorded from MFC and DS without any injection. (*B*) Reward acquisition probabilities in Day 1 and Day 2 are shown in control (black) and muscimol (red) groups. Each symbol shows data from a rat. Colored (black or red) symbols indicate the individual rats that showed significantly different probabilities (χ2 test, *P* < 0.05) between Day 1 and Day 2. Arrowhead indicates the rat shown in c. The differences in reward acquisition probabilities between Day 1 and Day 2 were compared between groups by two-sample *t*-test. (*C*) An example of impaired adaptive choice behavior of a muscimol-injected rat is shown on Day 1 (*top*, no injection) and Day 2 (*bottom*, muscimol injection). (*D*) Mean number of choice patterns per block on Day 1 (*left*) and Day 2 (*right*) in control group (*left* panel) and muscimol group (*right* panel). R: rewarded in last trial, U: unrewarded in last trial, Rp: repeat in current trial, Sw: switch in current trial. (*E*) Firing patterns of DS cells were compared (Mann–Whitney U test) in the muscimol group in terms of mean firing rate (*left*) and inter-spike interval (*right*) between Day 1 (*n* = 130, orange) and Day 2 (*n* = 77, red). Circles indicate the firing rates of individual DS neurons. (*F*) Time evolution of the fraction of (*top*) choice-modulated and (*bottom*) outcome-modulated DS cells (two-way ANOVA, 200-ms-long sliding-by-20-ms bins, *P* < 0.05) on Day 1 (orange) and Day 2 (red). Blue bars indicate bins showing statistical significance of difference in cell fractions between Day 1 and Day 2 (χ2 test, *P* < 0.05). Horizontal lines indicate the analysis window for calculation of selectivity index about choice (purple) and outcome (green) in G. (*G*) Comparison of selectivity index of DS neurons for choice (*left*) and outcome (*right*) information among 4 rats under the muscimol injection condition (Day 2). Arrowhead indicates the rat which improved performance at Day 2. Horizontal lines and error bars show the mean and s.e.m., respectively. Statistical significance was assessed by one-way ANOVA (*P* value is shown at top) followed by Tukey–Kramer test (asterisk indicates significance, *P* < 0.05).

To confirm the contributions of inter-trajectory correlations between MFC and DS to adaptive control, we examined the effect of bilateral MFC inhibition on the choice behavior and DS-cell activity in 5 rats (muscimol group) (Fig. 7*A*, Materials and Methods). Another 10 rats were examined for the choice behavior without bilateral MFC inhibition (control). The muscimol injection induced variable choice patterns in all the muscimol-treated rats (F test, *F* = 16.95, *P* = 0.0179). After the injection (Day 2), one rat showed no significant changes in behavior, but four rats exhibited significantly different reward acquisition probabilities from those prior to the injection (Day 1) (χ2 test, *P* < 0.05). The reward acquisition behavior was impaired in three rats among the four while it was improved in another rat (Fig. 7*B*). Figure 7*C* shows an example of impaired task performance, in which a rat reduced “repeat” behavior for rewarded outcomes but increased such behavior for unrewarded outcomes (Fig. 7*D*). Indeed, the fractions of “repeat” and “switch” were significantly different between Day 1 and Day 2 in the muscimol group (two-way ANOVA, *F*_1,4_ = 5.774, *P* = 0.021) but not in the control group (*F*_1,9_ = 0.248, *P* = 0.620), and changes in the reward acquisition probabilities between Day 1 and Day 2 were statistically different between the two groups (two-sample *t*-test, *P* = 0.0472). Because the number of trials was not significantly different between Day 1 (mean ± SD = 731 ± 86 trials) and Day 2 (740 ± 96 trials) in the muscimol group (unequal variance *t*-test, *P* = 0.877) and because these numbers were not significantly different from those of the control group, the behavioral difference between the two groups was unlikely attributed to the attenuation of their motivations for the task.

The inhibition of MFC diminished neuronal activities and their selective modulations in DS. The average firing rate of DS cells were significantly lower (Mann–Whitney U-test, *P* = 0.0157) and the median of inter-spike intervals was significantly longer (Mann–Whitney U-test, *P* = 0.0051) on Day 2 (*n* = 77) than on Day 1 (*n* = 130) (Fig. 7*E*), suggesting an attenuated excitatory drive or an enhanced inhibition on DS cells. The MFC inhibition also altered the task-related activities of DS cells, especially those encoding choices and outcomes (two-way ANOVA, *P* < 0.05). The proportion of choice-modulated cells around and after choice responses was significantly smaller on Day 2 than on Day 1 (χ2 test, *P* < 0.05) (Fig. 7*F*, top), and so was the proportion of outcome-modulated cells during the late outcome period (more than 2 s after a choice response). However, this was not the case for the earlier outcome period (Fig. 7*F*, bottom). We addressed the question whether there were any neuronal correlates of behavior in the rat with improved performance after muscimol injection (Fig. 7*B*). We calculated selectivity index for each DS neuron during the period showing an increased fraction of selective neurons in Figure 7*F* (see section Materials and Methods). Selectivity index for outcome was not significantly different among the four rats (one-way ANOVA, *P* = 0.569) despite that they displayed different reward acquisition probabilities (Fig. 7*G*). By contrast, selectivity index for choice was significantly different among these rats (one-way ANOVA, *P* = 0.000625). In particular, selectivity index for choice tended to be higher in the rat with improved performance than in other rats with impaired performance (Tukey–Kramer test, *P* < 0.05) (Fig. 7*G*).

Results of muscimol injection suggest that cortical areas other than the MFC also contributed to the present adaptive choice behavior. Since injection needles were placed in the MFC region that frequently contained the corticostriatal neurons retrogradely labeled by Fluoro-Gold (Fig. S8), the pharmacological inactivation of MFC also affected activity in DS, diminishing the firing rates and proportion of selectively activated DS neurons. However, the inactivation of MFC did not completely suppress activity in DS. Related to this, DS neurons showed a stronger sequential property than MFC neurons, which may result from the integration of synaptic inputs from multiple cortical areas to DS. Taken together, our results indicate that neuronal activation in the MFC partly but strongly regulates the neural coding of choice- and outcome-related information in the DS.

## Discussion

In this study, we revealed a parallel evolution of MFC and DS neural trajectories during outcome-based choice behavior. These trajectories initially encoded a current choice and later represented the outcome of the choice response. The amount of information carried by the trajectories was not significantly different between MFC and DS, suggesting that these regions share a large portion of task-related information during the task. Furthermore, cross-area spike coincidences in the millisecond range were enhanced when the trajectories were also highly correlated between the two regions in rewarded trials, suggesting that the parallel trajectory evolution in MFC and DS actively participates in coordinating the behavioral task.

The strong similarity in neural coding in MFC and DS questions the conventional view of activity selection by the basal ganglia. Along the frontal cortex-basal ganglia axis, the MFC-DS channel is thought to be the stage to process action selection based on past outcomes (Nambu 2008; Hikosaka and Isoda 2010). A widely hypothesized mechanism for this process is that neural ensemble in DS selectively gates the necessary information (or suppresses the unnecessary information) received from MFC. Our results, however, are unlikely to support this hypothesis because neural populations in MFC and DS preserve similar amounts of information about choices as well as outcomes and their trajectories evolve quite similarly in the two regions (Fig. 4).

The parallel trajectory evolution in MFC and DS during the present outcome-based decision making is consistent with a previous finding demonstrating a simultaneous and correlated activation of time-dependent ramping activity in MFC and DS during a temporal judgment task (Emmons et al. 2017). Given that MFC innervates DS unidirectionally, an interpretation of our results is that MFC cells exhibit choice-related signals prior to DS cells and then sequentially convey these signals to DS. This was suggested in previous studies (Sul et al. 2011; Ma et al. 2014). However, our data do not seem to support this interpretation because choice-related signals concomitantly emerge in MFC and DS. The discrepancy may be attributed to differences in experimental conditions. Unlike the other studies, we recorded neuronal activities in a head-restrained condition, did not change the probability of reward delivery during the task, and directly compared simultaneous neuronal activations between MFC and DS.

Our findings suggest that MFC and DS cooperate on processing task-relevant information through the temporal coherence of neural activity at both population (neural trajectories) and single-cell (spike synchrony) levels. A previous study in freely moving rats demonstrated oscillatory synchronization of the local field potentials at 5–13 Hz across the motor cortex, DS and substantia nigra pars reticulata (an output terminal of the basal ganglia) and propagation of spindle-like spike-and-wave oscillations along this cortico-basal ganglia pathway. Spikes of individual neurons were phase-locked to the oscillatory local field potentials and synchronized within the cortical-basal ganglia network (Dejean et al. 2007). Synchronized neuronal discharges are thought to enable a reliable information transmission (Salinas and Sejnowski 2001; Buzsáki and Schomburg 2015), and the temporal correlation between neural ensembles in the frontal cortex-basal ganglia network could emerge through the learning of the decision behavior. Actually, synchronous spiking was coordinated between certain MFC and DS cells when neural trajectories in these areas were strongly correlated in the well learned rats, supporting the hypothesis (Fig. 6). In accordance with this view, during skill learning, neural ensembles in both motor cortex and DS are known to develop spike correlations (Costa *et al*., 2004; Santos et al. 2015) and precise temporal spiking patterns (Koralek et al. 2013; Lemke et al. 2019).

How was the precise spike synchrony generated between the cortical and subcortical regions recorded in this study? There are several possible explanations based on neural circuit structures. Medium spiny cells in DS receive monosynaptic excitatory inputs from ipsilateral and contralateral MFC (Wilson 1986) and we indeed confirmed that bilateral MFC neurons were retrogradely labeled after Fluoro-Gold injection in DS in the present study (Fig. S8*A*). In addition, MFC cells’ output projects to both ipsilateral and contralateral MFC and DS (Wilson 1987; Reiner et al. 2003). One possibility is that these direct projections from MFC induce spike synchrony in DS. In this case, MFC cells should fire prior to the firing of DS cells, but this was not the case in the CCGs between these cells (Fig. 6). Another possibility is that the firing of MFC and DS cells was driven by a common input from other corticostriatal neurons in the MFC (Shepherd 2013; Kawaguchi 2017). Since excitatory connections are abundant between MFC corticostriatal pyramidal cells (Morishima and Kawaguchi 2006), synchronous discharges of MFC neurons can generate large EPSPs in their postsynaptic target cells in MFC and DS, increasing the probability of synchronous firing between MFC and DS.

Alternatively, other cortical or subcortical areas could provide common drives. Indeed, we observed synchronous firing in which DS neurons sometimes discharged slightly earlier (submillisecond order) than MFC neurons (Fig. 6D), which cannot be explained by the feedforward corticostriatal circuit with non-reciprocal corticostriatal connections (MFC- > DS). Because MFC (Reep et al. 1990) and DS (Cheatwood et al. 2003; Reep et al. 2003) receive afferent connections from higher-order cortical areas such as the ventrolateral OFC and PPC, these areas may evoke synchronous firing of MFC and DS cells. Actually, OFC and PPC are implicated in goal-directed decision-making (Gremel and Costa 2013b; Erlich et al. 2015). For instance, optogenetic inhibition of PPC corticostriatal neurons altered a history-dependent choice bias in decision making (Hwang et al. 2019). Among subcortical area, certain thalamic nuclei including the ventrolateral, mediodorsal, and intralaminar thalamic nuclei commonly project to rostral AGm and dorsocentral striatum (Hicks and Huerta 1991; Reep and Corwin 1999; Cheatwood et al. 2003). In a recent work, thalamostriatal inputs could activate medium spiny neurons in DS during reward conditioned behavior even if the secondary motor cortex was bilaterally inhibited by an optogenetic method (Lee et al. 2019).

In the present outcome-based action selection, bilateral MFC inactivation attenuated, but did not completely eliminate, choice and outcome representations in DS cells (Fig. 7), suggesting that brain regions other than MFC also drive DS cells. This explanation also seems to be consistent with the sequential property of DS neurons (Fig. 3*F*), which indicates a heavier commitment of DS than MFC to tracking the temporal and/or operational flow of a behavioral task. Such a commitment likely requires the integration as well as selection of dispersed behaviorally relevant information. We note that the difference in sequentiality does not necessarily conflict with the observation that both areas maintain about equal amount of behavioral information. Adaptive reward-based action selection is thought to recruit network mechanisms involving multiple cortical and subcortical regions. Further recordings from multiple regions involving MFC, OFC, PPC, thalamic nuclei and DS are required to distinguish between the different causes of spike synchrony between MFC and DS.

It was recently shown in the three-layered cortex of the reptile that just a few spikes of single pyramidal neurons can trigger a cascade of firing sequences of neuron ensembles (Hemberger et al. 2019), as was suggested by theoretical studies (Diesmann et al. 1999; Salinas and Sejnowski 2001). If a similar cascade of firing sequences is triggered in the cortico-basal ganglia pathway during the parallel evolution of frontal cortical and striatal neural trajectories, such spike sequences may not only enhance reliable information transmission (i.e., spikes) from the cortex to the basal ganglia, but also may contribute to regulating the gain of synaptic plasticity together with modulations by dopaminergic afferents (Pawlak and Kerr 2008; Shen et al. 2008). Clarification of this interesting possibility is open for future studies.

We also observed suppressive activity in DS after reward delivery (Fig. 3*E*), as was reported previously (Shin et al. 2018). One possible cause of this suppression is striatal GABAergic interneurons which strongly inhibit the medium spiny neurons (Koós and Tepper 1999; Lee et al. 2017). Not only glutamatergic inputs from the cortex and thalamus but also neuromodulators such as dopamine and acetylcholine depolarize GABAergic interneurons in the striatum (Bracci et al. 2002; Centonze et al. 2002; Koós and Tepper 2002). The role of this inhibition in decision making has yet to be explored.

## Funding

The Grants-in-Aid for Scientific Research (KAKENHI) from MEXT (no. 24700345 to T.H., nos. 18H05213 and 19H04994 to T.F.).

## Notes

We thank T. Sharp and T. Takekawa for their advice and supports for initial analysis on our data. We are grateful to M. Gilson, P. Goncalves, J. Igarashi, Y. Isomura, T. Kurikawa, and M. Tatsuno for their valuable comments on our results. We thank J. Wickens for his critical reading of our manuscript and Y. Goda for her advices. We thank I. Alsolami for his useful suggestion on data analysis. *Conflict of Interest:* The authors declare no competing financial interests.

## Author contributions

T.H. and T.F. designed the project. T.H. and R.H. conducted experiments. T.H. analyzed data. T.H. and T.F. wrote the manuscript.

## Supplementary Material

20210212_SupplementaryFigures_bhab091
