# Supplementary material for: Concomitant Processing of Choice and Outcome in Frontal Corticostriatal Ensembles Correlates with Performance of Rats
Source: Cereb Cortex. 2021 Apr 29;31(9):4357–75. doi: 10.1093/cercor/bhab091 (PMC8328202; doi:10.1093/cercor/bhab091)
Supplement: 20210212_SupplementaryFigures_bhab091 [file 20210212_supplementaryfigures_bhab091.pdf]

Figure S1

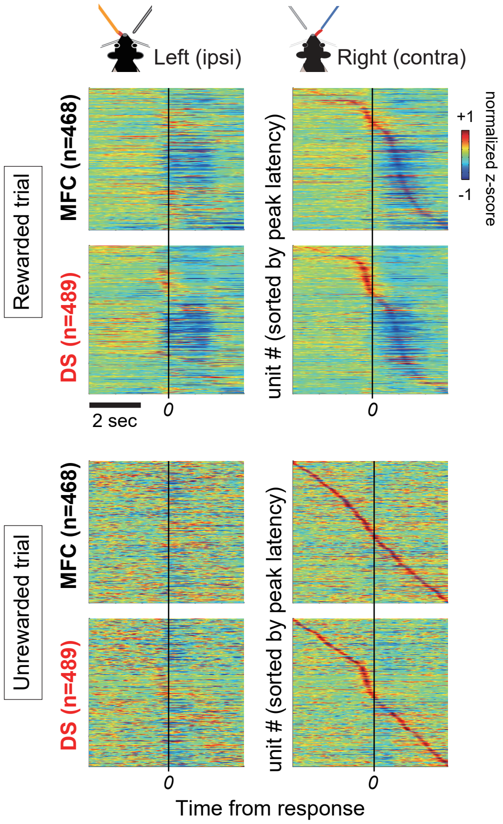

Figure S1. Collective PETHs sorted by peak latency at right choice trials.

The same collective PETH as in Fig. 3E is presented in the order of units sorted by the latencies of maximum absolute responses in rewarded (*top*) and unrewarded (*bottom*) right choice trials.

Figure S2

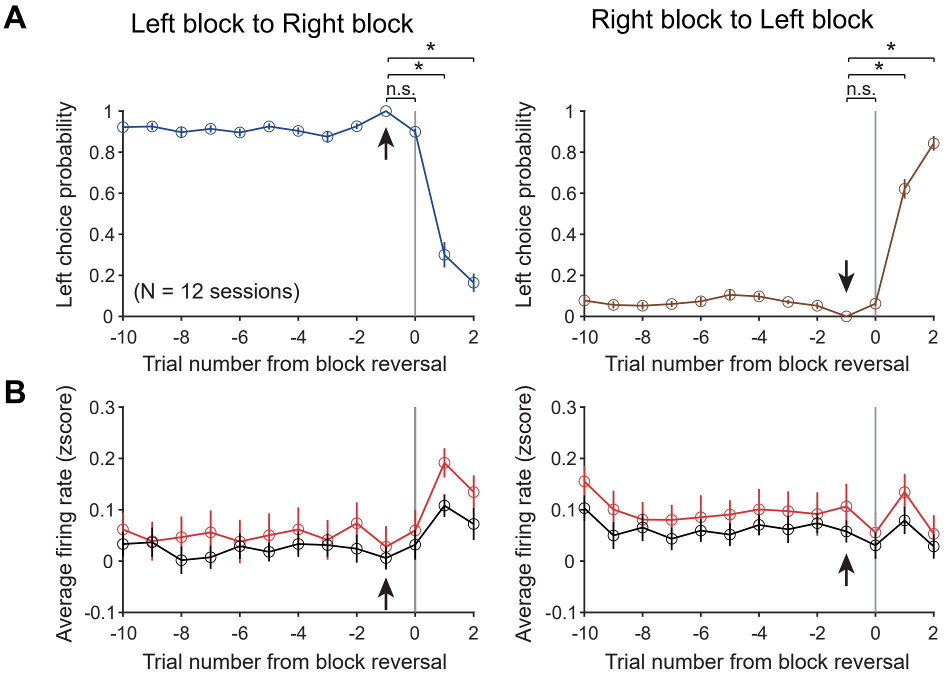

Figure S2. Trial series of choice probability and collective firing rates over reward block reversal. (A) Averaged Left choice probability in 12 recording sessions shown in Figure 3E-G. Mean left choice probability is plotted when reward position is reversed from Left to Right spout (*left*) and from Right to Left spout (*right*). Zero indicates the first trial after block reversal. **(B)** Averaged collective firing rates during 1-s before choice response in MFC (black) and DS (red) recorded in the12 recording sessions. Error bars show s.e.m.. Black arrow indicates the last (10th) rewarded trial. Asterisks indicate statistical significance in comparison with data in the last rewarded trial (one-way ANOVA followed by Tukey-Kramer test, $p<0.05$).

Figure S3

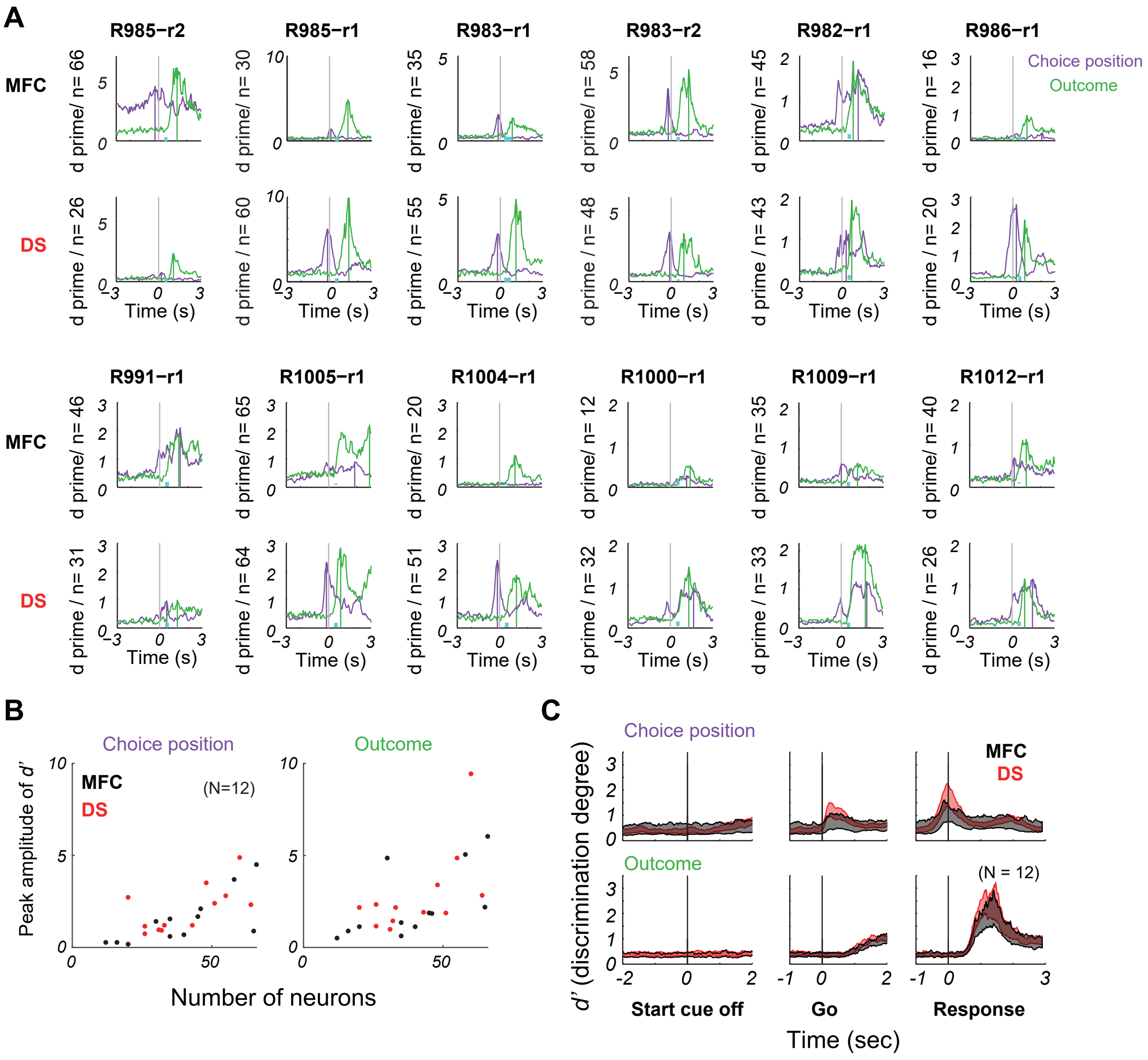

Figure S3. Degree of discrimination by population activity at individual sessions. **(A)** Time evolution of discrimination degree (*d'*) on choice (purple) and outcome (green) axes around choice responses. Vertical line indicates the peak time of *d'* and cyan bar the interval of reward delivery. **(B)** Correlations between peak *d'* values and the number of neurons in population data. **(C)** Group data of *d'* on choice and outcome axes in MFC (black) and DS (red). Histograms were aligned at Start cue offset, Go cue onset, and Response execution.

Figure S4

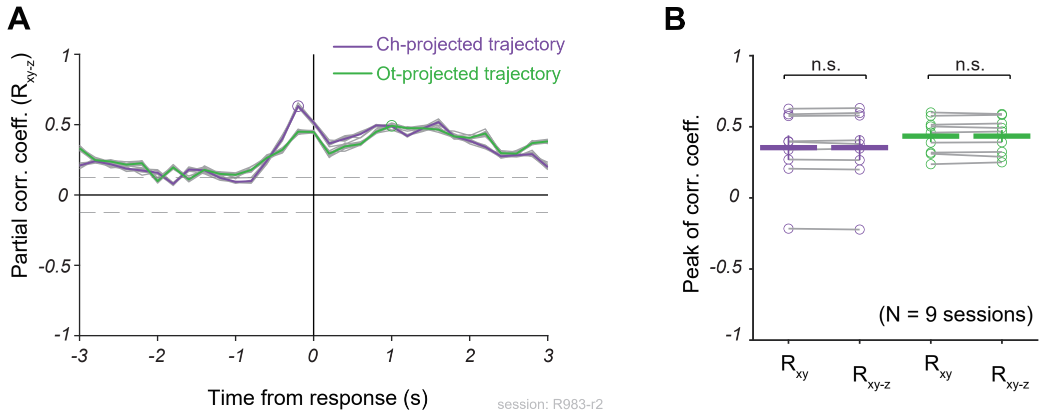

Figure S4. Partial correlation of neural trajectory between MFC and DS . (A) Time series of partial correlation coefficients ($R_{xy\text{-}z}$) was calculated between MFC and DS projected trajectories shown in Figure 4B. Purple and green lines represent the mean partial correlation coefficients, and gray solid lines show individual partial correlation coefficient calculated from randomly sampled 431 trials. Circles indicate the peak of the correlation coefficients. Horizontal gray dashed lines represent a criterion for statistical significance ($p = 0.01$, $t = 2.588$, $df = 430$). **(B)** Comparison of peak of partial correlation coefficients ($R_{xy\text{-}z}$) with peak of correlation coeffeficients (R_{xy}) in Ch-projected (purple) and Ot-projected (green) trajectories in 9 sessions. Statistical significance is assessed by paired t-test ($p < 0.05$).

Figure S5

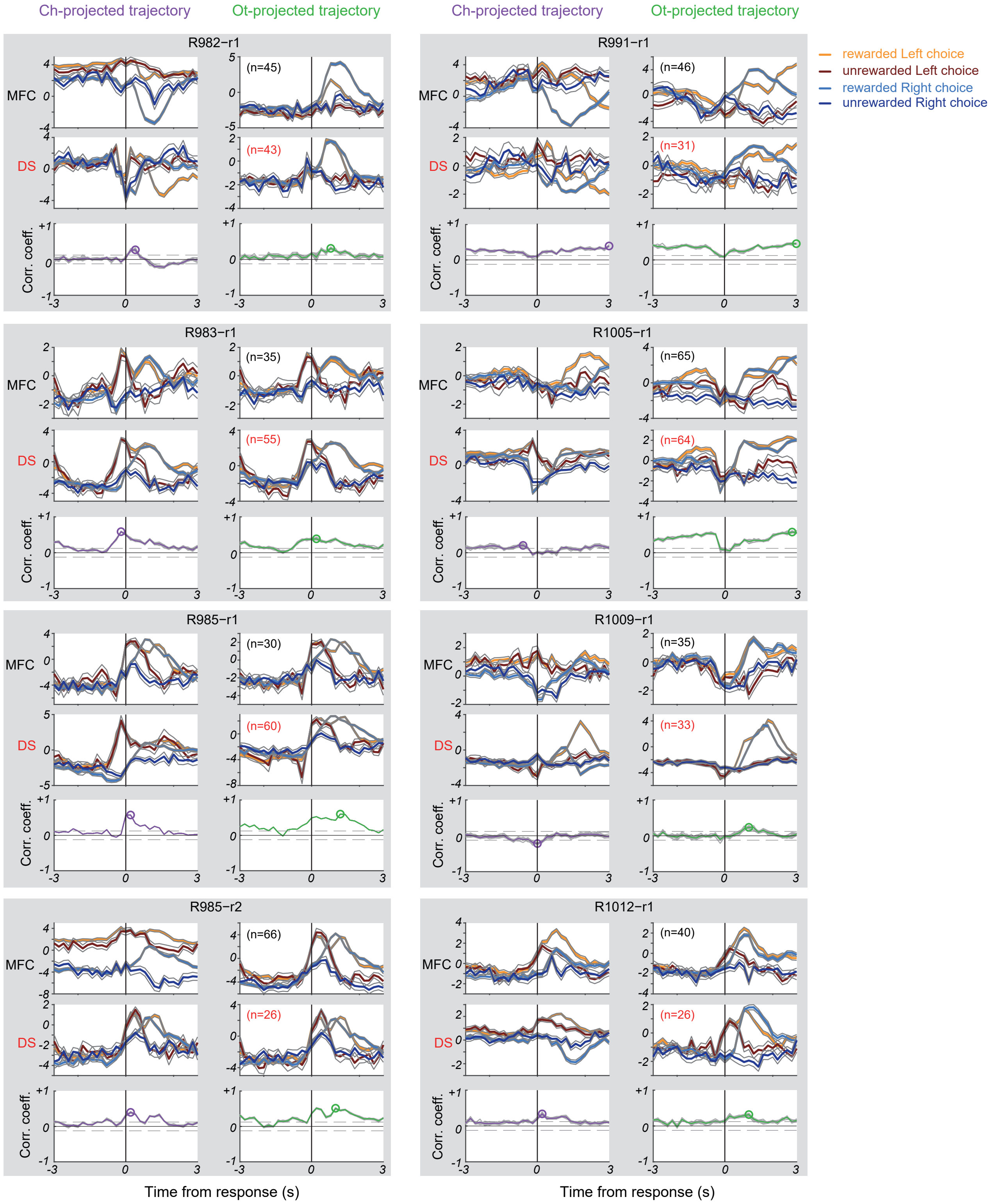

Figure S5. MFC and DS trajectories and their session-by-session correlations.
Each gray box represents data from individual session. Within the box, neural trajectories in MFC (*top*) and DS (*middle*) on choice (*left*) and outcome (*right*) axes are shown. Color codes indicate Left rewarded trials (orange), Left unrewarded trials (brown), Right rewarded trials (light blue) and Right unrewarded trials (dark blue). Thin lines represent s.e.m.. (*bottom*) Time evolution of correlation coefficients between MFC and DS trajectories. Gray solid lines show the correlation coefficients calculated with randomly sampled 431 trials (repeated 10 times). The averaged correlation coefficients between MFC and DS are displayed for Ch-projected (*left*, purple) or Ot-projected (*right*, green) trajectories. Circles indicate the times of highest correlation coefficient. Horizontal gray dashed lines represent a criterion for statistical significance ($p = 0.01$, $t = 2.588$, $df = 430$).

Figure S6

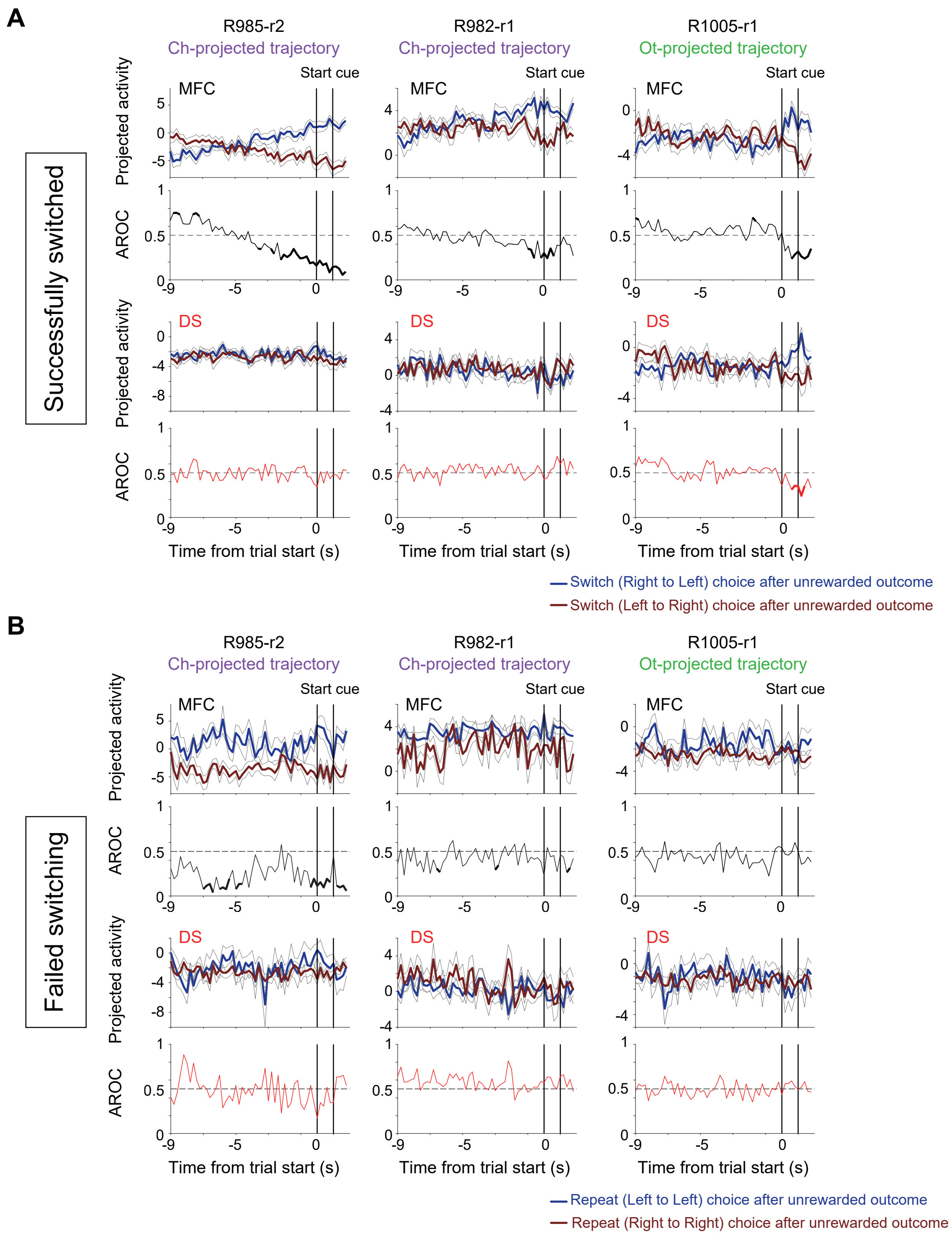

Figure S6. Comparison of neural trajectories during inter-trial interval (ITI) period post unrewarded episode between when rats successfully switched choice and when rats failed to switch choice. Two neural trajectories during ITI period post unrewarded episode in 3 sessions (*left*: R985-r2; *middle*: R982-r1; *right*: R1005-r1) in MFC (1st row) and DS (3rd row) when rats successfully switched **(A)** and failed to switch **(B)**. Black thin lines in 1st and 3rd rows denote s.e.m.. Differences in the two trajectories were quantified by AROC values in MFC (2nd row, black) and DS (4th row, red). A gradual shift from high values (> 0.5) to low values (< 0.5), suggests that the trajectories evolved to the opposite side of the corresponding hyperplane (as shown at 2nd row for R985-r2 in **A**). Statistical significance of AROC values is assessed by Mann-Whitney U test ($p < 0.05$) and represented by thick lines. Two vertical lines show the duration of start cue presentation.

Figure S7

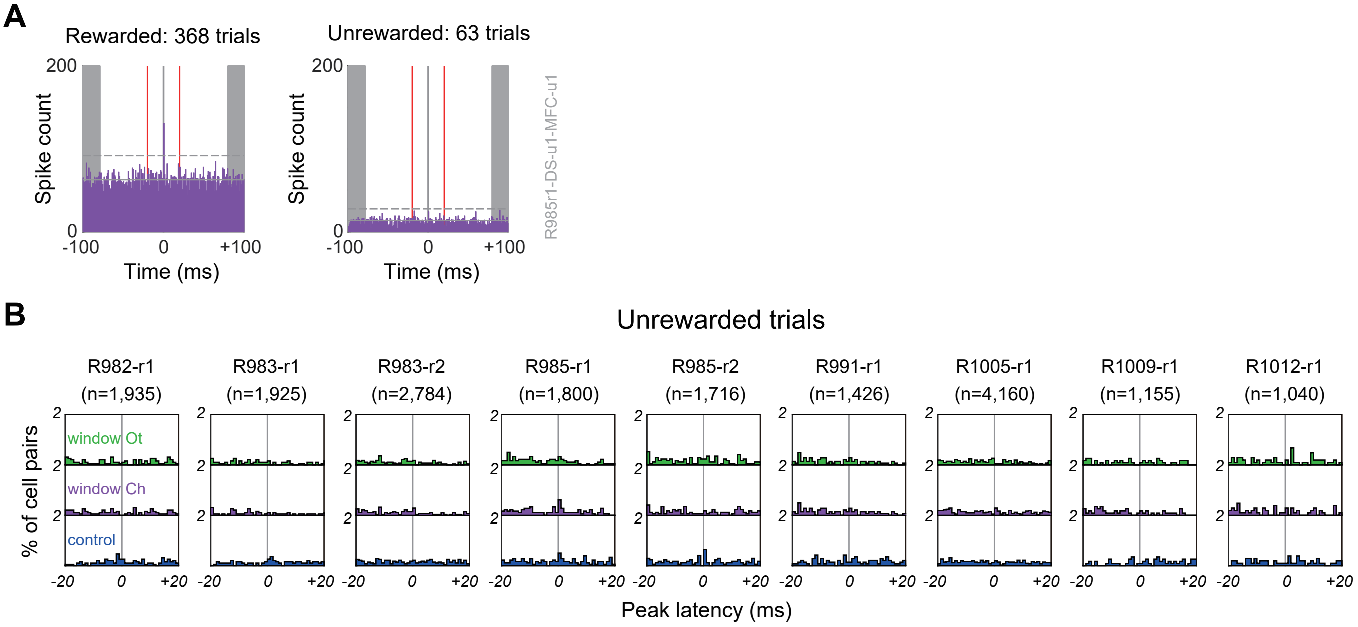

Figure S7. Cross correlaton of spike timeing between MFC and DS cells in unrewarded trials.

(A) CCGs of the MFC-DS cell pair shown in Figure 6A in rewarded (*left*) and unrewarded (*right*) trials. **(B)** Proportion of MFC-DS cell-pairs that displayed an excess amount of spike coincidences in unrewarded trials is plotted for the 9 sessions against CCG peak time lags. The numbers of total cell-pairs are given in parentheses. CCG peak values of cell-pairs in windows Ch (purple) and Ot (green) are compared with those in task-event irrelevant windows (blue).

Figure S8

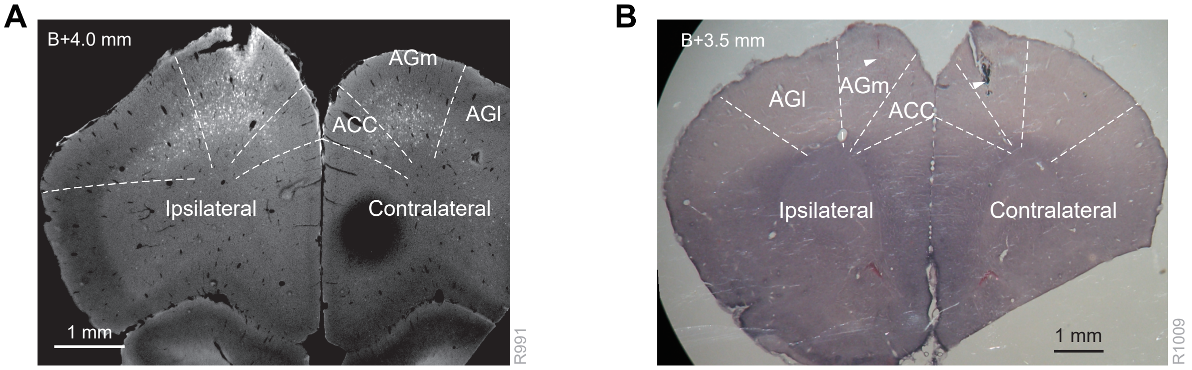

Figure S8. Bilaterally Fluoro-Gold labelled MFC neurons and muscimol injections sites in MFC.

(A) Retrogradely Fluoro-Gold labelled neurons in both ipsilateral and contralateral MFC (particularly in rostral AGm) to Fluoro-Gold injection site (DS in left hemisphere as shown in Figure 2B). This brain section is shown in Figure 2B. Black spot in the contralateral side is an area bleached during *post hoc* microscopyic observations. AGl: agranular lateral area, ACC: anterior cingulate cortex. **(B)** *Post hoc* confirmation of bilateral muscimol injection sites. The needle tracks (presented by arrowheads) were confirmed in AGm.
